# Porphyrin-based donor–acceptor COFs as efficient and reusable photocatalysts for PET-RAFT polymerization under broad spectrum excitation†

**DOI:** 10.1039/d1sc05379e

**Published:** 2021-11-26

**Authors:** Yifan Zhu, Dongyang Zhu, Yu Chen, Qianqian Yan, Chun-Yen Liu, Kexin Ling, Yifeng Liu, Dongjoo Lee, Xiaowei Wu, Thomas P. Senftle, Rafael Verduzco

**Affiliations:** Department of Materials Science and NanoEngineering, Rice University Houston Texas 77005 USA; Department of Chemical and Biomolecular Engineering, Rice University Houston Texas 77005 USA tsenftle@rice.edu rafaelv@rice.edu; Department of Chemistry, Rice University Houston Texas 77005 USA; CAS Key Laboratory of Design and Assembly of Functional Nanostructures, Fujian Provincial Key Laboratory of Nanomaterials, Fujian Institute of Research on the Structure of Matter (FJIRSM), Chinese Academy of Sciences Fuzhou 350002 China xmwuxiaowei@fjirsm.ac.cn; Xiamen Key Laboratory of Rare Earth Photoelectric Functional Materials, Xiamen Institute of Rare Earth Materials (XMIREM), Haixi Institutes, Chinese Academy of Sciences Xiamen 361021 China

## Abstract

Covalent organic frameworks (COFs) are crystalline and porous organic materials attractive for photocatalysis applications due to their structural versatility and tunable optical and electronic properties. The use of photocatalysts (PCs) for polymerizations enables the preparation of well-defined polymeric materials under mild reaction conditions. Herein, we report two porphyrin-based donor–acceptor COFs that are effective heterogeneous PCs for photoinduced electron transfer-reversible addition–fragmentation chain transfer (PET-RAFT). Using density functional theory (DFT) calculations, we designed porphyrin COFs with strong donor–acceptor characteristics and delocalized conduction bands. The COFs were effective PCs for PET-RAFT, successfully polymerizing a variety of monomers in both organic and aqueous media using visible light (*λ*_max_ from 460 to 635 nm) to produce polymers with tunable molecular weights (MWs), low molecular weight dispersity, and good chain-end fidelity. The heterogeneous COF PCs could also be reused for PET-RAFT polymerization at least 5 times without losing photocatalytic performance. This work demonstrates porphyrin-based COFs that are effective catalysts for photo-RDRP and establishes design principles for the development of highly active COF PCs for a variety of applications.

## Abstract 2

Porphyrin-based donor–acceptor COFs are effective heterogeneous photocatalysts for photoinduced electron transfer-reversible addition–fragmentation chain transfer (PET-RAFT), including for aqueous polymerizations and under red-light excitation.

## Graphical abstract

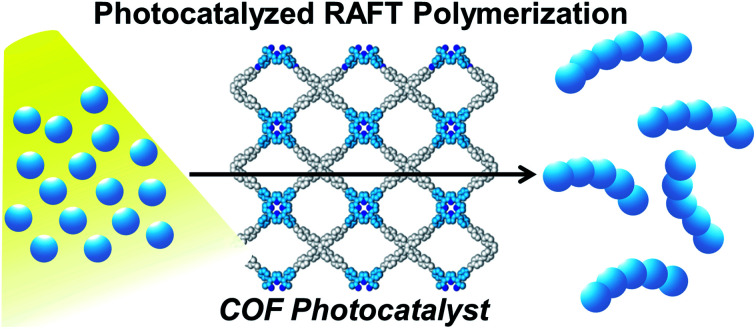

## Introduction

Covalent organic frameworks (COFs) are an emerging class of porous crystalline organic materials featuring high surface areas and excellent thermal and chemical stabilities.^1–3^ Their use as photocatalysts (PCs) is particularly intriguing because they have a high surface area for adsorption, tunable pore and surface functionalities, and variable optical and electronic properties determined by the properties of the COF building blocks.^4–6^ Prior work has taken advantage of this tunability to increase interactions with a specific molecule or substrate, and their optical and electronic properties can also be tailored for a specific photocatalytic application.^4,7^ For example, the use of donor–acceptor building blocks increases the lifetime of excitonic carriers in a charge-transfer state,^8–10^ and the bandgap can be tuned to increase visible light adsorption.^11^ Furthermore, the highly crystalline and conjugated structure can accelerate charge transport,^5^ and the conjugated structure in both the in-plane and stacking directions facilitates high charge carrier mobility.^11^ In light of these attractive features, COFs have been explored as PCs for a variety of reactions and applications, including hydrogen evolution,^12,13^ carbon dioxide reduction,^14^ degradation of organic pollutants,^15^ and other organic transformations.^16–18^

Recently, there has been significant interest in the development of PCs for reversible deactivation radical polymerization (RDRP) methodologies,^19–21^ such as atom transfer radical polymerization (ATRP)^22^ and reversible addition–fragmentation chain-transfer (RAFT) polymerization.^23^ The use of PCs for polymerizations offers access to well-defined polymeric materials with complex architectures under mild reaction conditions,^20^ and heterogeneous PCs offer added advantages such as easy separation, recycling, and excellent purity of the final product free of catalysts contaminants.^24^ Examples include small molecular PCs immobilized on heterogeneous supports,^25^ as well as inorganic semiconductors such as perovskites,^26^ CdSe quantum dots,^27,28^ Bi_2_O_3_,^29^ metal–organic frameworks (MOFs),^30–32^ and plasmonic Ag_3_PO_4_.^33^ Especially, MOFs and their nanosheets have been utilized in photoinduced 3D printing, exhibiting excellent oxygen tolerance and the ability to print complex objects.^30,31^ However, COFs represent a promising but poorly explored class of heterogeneous and metal-free PCs for photo-RDRP. Hou and coworkers reported a COF-mediated photo-ATRP using copper catalysts.^34^ The same group also demonstrated the use of fully-π conjugated COFs as PCs to mediate ATRP (with ppm-level copper)^35^ and pyrene-based COFs for RAFT^36^ under white-light irradiation. Cui and coworkers reported *N*,*N*-diaryl dihydrophenazine-based 2D and 3D COFs that promoted photoinduced radical ring-opening polymerization of vinylcyclopropanes,^37^ achieving controlled molecular weight and low molecular weight dispersities. These studies demonstrated that COFs could be effective PCs for RDRP, but we still lack an understanding of how to properly design COFs for RDRP, such as selecting the proper building blocks and designing a COF with proper band level alignment and optical properties. It is also unclear whether these COF catalysts can be effective with long-wavelength visible light, and whether the performance can be further enhanced through increased donor–acceptor interactions.

**Scheme 1:**
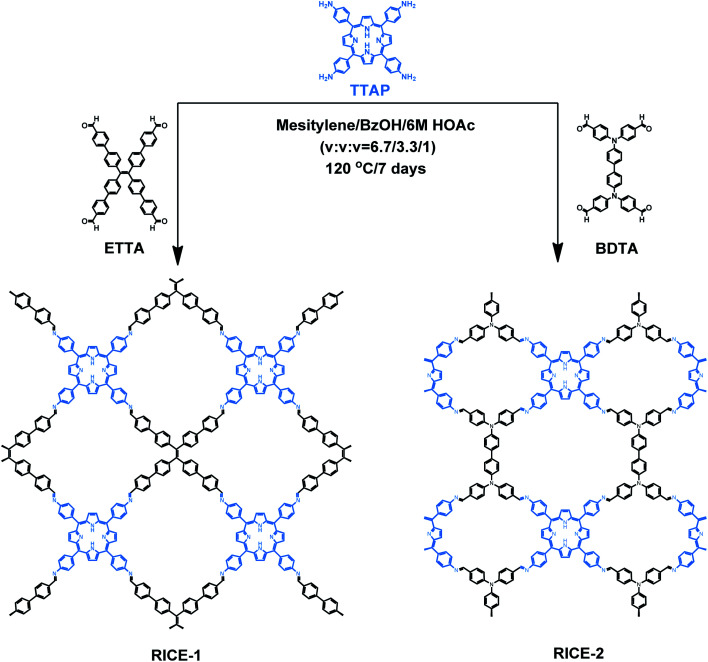
Synthesis of porphyrin COFs RICE-1 and RICE-2. COFs were synthesized in mesitylene and benzyl alcohol (BzOH) with acetic acid (HOAc) as the catalyst.

Herein, we report the design, synthesis, and use in PET-RAFT of two two-dimensional (2D) porphyrin-based COFs. We synthesized highly crystalline COFs, termed RICE-1 and RICE-2, using a porphyrin derivative (5,10,15,20-tetrakis(4-aminophenyl)porphyrin, TTAP) with a *C*_4_ geometry and two aldehyde monomers (4′,4′′,4′′′,4′′′′-(ethene-1,1,2,2-tetrayl)tetrabiphenyl-4-aldehyde, ETTA), and (4,4′,4′′,4′′′-([1,1′-biphenyl]-4,4′-diylbis(azanetriyl))tetrabenzaldehyde, BDTA) with tetrapopic C2/D2 geometry as building units (Scheme 1). The selection of these node and linker monomers was guided based on density functional theory (DFT) calculations, which indicated that this combination of monomers would produce strong donor–acceptor characteristics in the final COFs, delocalization of the conduction band in the COF, and proper energy level alignment for electron transfer to RAFT chain transfer agents (CTA). The synthesized COFs exhibited excellent performance in PET-RAFT polymerization of a variety of monomers in various solvents under a broad spectrum of irradiation (*λ*_max_ from 460 to 635 nm), producing polymers with tunable molecular weight (MWs) and narrow molecular weight dispersities (*Đ*). The heterogeneous COF PCs also showcased good recyclability and could be reused for PET-RAFT polymerization at least 5 times without losing photocatalytic performance. This work demonstrates porphyrin-based COF that are effective catalysts for photo-RDRP and, more broadly, demonstrates how DFT can guide the proper selection of building blocks to design highly active COF PCs for a variety of applications.

## Results and discussion

As a starting point for the rational design of metal-free COF PCs that can catalyze PET-RAFT under visible light irradiation, we chose the porphyrin derivative TTAP. Porphyrins have previously been used in PET-RAFT polymerizations and are capable of absorbing low-energy visible light.^38^ We also targeted a COF with strong donor–acceptor characteristics that could both promote spontaneous charge separation and enhance charge carrier mobility, potentially resulting in metal-free COFs with excellent photocatalytic properties.^9^ Using computational DFT analysis (Fig. S1†), we found that the linkers ETTA and BDTA (Scheme 1) could form COFs with strong donor–acceptor characteristics when paired with TTAP. The lowest unoccupied molecular orbital (LUMO) of TTAP (−2.19 V *vs.* standard hydrogen electrode, SHE) is more negative than that of ETTA (−1.71 V_SHE_) and BDTA (−1.93 V_SHE_), while the highest occupied molecular orbital (HOMO) of TTAP (0.38 V_SHE_) is less positive than that of ETTA (1.58 V_SHE_) and BDTA (1.50 V_SHE_). These results suggest that TTAP could serve as an efficient electron donor when paired with EETA and BDTA and that the resulting structures would have strong donor–acceptor characteristics.

As shown in Scheme 1, RICE-1 and RICE-2 were prepared using TTAP with ETTA and BDTA, respectively, in a mixture of mesitylene, BzOH, and 6 M HOAc (v/v/v = 6.7 : 3.3 : 1) at 120 °C for 7 days. The products were thoroughly washed and dried overnight at 60 °C under vacuum, affording RICE-1 and RICE-2 with isolated yields around 69% and 82%, respectively. Through Fourier transform infrared (FT-IR) spectroscopy, we verified the presence of C=N stretching bands at 1621 cm^−1^ for RICE-1 and 1617 cm^−1^ for RICE-2, along with the disappearance of C=O and N–H stretching bands present for the starting materials (Fig. S2 and S3†). X-ray photoelectron spectroscopy (XPS) survey scans exhibited peaks near 284, 397 and 532 eV, which we attributed to the C1s, N1s, and O1s binding energies,^39^ respectively (Fig. S4 and S5†). Scanning electron microscopy (SEM) images of both COFs displayed aggregated macroparticles morphology (Fig. S6†). Thermogravimetric analysis (TGA) revealed that both COFs are thermally stable up to 500 °C, indicative of their excellent thermal stabilities (Fig. S7†).

**Fig. 1:**
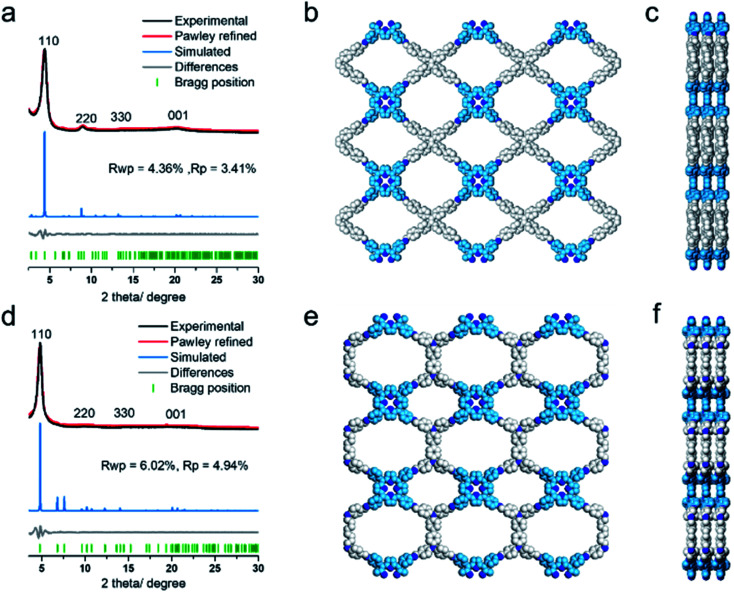
(a) and (d) PXRD patterns of RICE-1 (a) and RICE-2 (d) with the experimental profiles in black, Pawley refinement in red, simulated AA stacking model in blue, their difference in grey, and the Bragg position in green. (b) and (c) Structural representations of RICE-1 (b) front view (c) side view. (e) and (f) Structural representations of RICE-2 (e) front view (f) side view.

The COF products displayed sharp diffraction peaks under powder X-ray diffraction (PXRD) analysis (Fig. 1). Using the Reticular Chemistry Structure Resource (RCSR),^40^ a number of plausible nets (cpq and hna *etc.*) and stacking models (eclipsed AA, staggered AB, *etc.*) were identified, and molecular simulations (Fig. 1, S8 and S9†) clearly showed that both COFs likely adopted cpq topologies with eclipsed AA stacking. PXRD of RICE-1 showcased a prominent 110 peak located at 4.34° along with weak peaks at 8.79, 13.11 and 20.15°, which corresponded to 220, 330 and 001 facets. RICE-2 exhibited a similar PXRD pattern to that of RICE-1 (Fig. 1d and S10† with enlarged PXRD), demonstrating the isoreticular 2D cpq net. We obtained unit cell parameters after Pawley refinements (space group: *P*222, *a* = 31.07 Å, *b* = 26.31 Å, *c* = 4.39 Å for RICE-1, *a* = 26.00 Å, *b* = 26.00 Å, *c* = 4.42 Å for RICE-2), and they were in good agreement with experimental ones as reflected in low residual values and small profile differences.

We further analyzed the porosity of both COFs by nitrogen sorption measurements at 77 K (Fig. S11†). Both COFs exhibited type I isotherms with an abrupt increase at low relative pressures (*P*/*P*_0_ < 0.01) characteristic of microporosity and indicating highly porous structures. The Brunauer–Emmett–Teller (BET) specific surface areas were determined to be 443.9 m^2^ g^−1^ for RICE-1 and 506.6 m^2^ g^−1^ for RICE-2. Pore size distributions of both COFs were calculated using nonlocal density functional theory (NLDFT) (Fig. S11b–d†). Both COFs possessed two types of pores (sizes of 1.4 nm and 1.6 nm for RICE-1 and 1.1 nm and 1.4 nm for RICE-2), and the pore sizes agreed well with the simulated structures (Fig. 1).

**Fig. 2:**
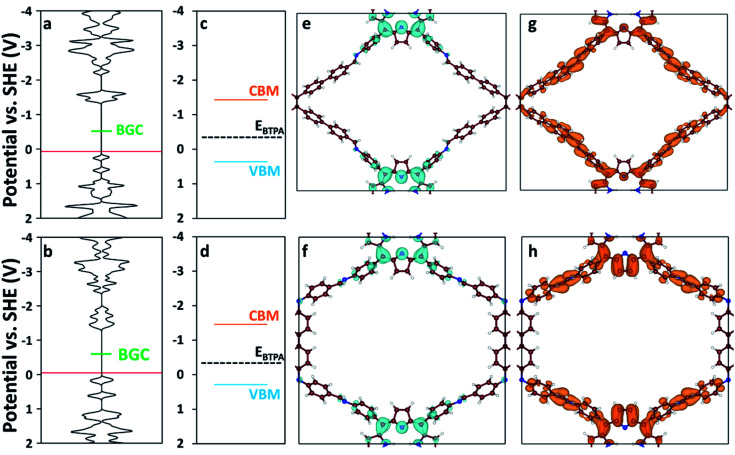
Density of states (DOS) of (a) RICE-1 and (b) RICE-2. The horizontal axis of (a–d) has units of states eV^−1^ cell^−1^. The red lines represent the highest occupied energy level and the green lines represent the position of the calculated BGC. Band diagrams of (c) RICE-1 and (d) RICE-2 were calculated from the BGC in (a) and (b), respectively, combined with the experimentally measured bandgap using eqn (S1) and (S2).† Orange, blue, and black-dashed lines represent CBM, VBM, and the position of BTPA redox potential (*E*_BTPA_), respectively.^23^ The orbital images of the VB edge (blue isosurfaces) of (e) RICE-1 (potential range: 0.45 V_SHE_ to −0.01 V_SHE_ in (a)) and (f) RICE-2 (potential range: 0.19 V_SHE_ to −0.13 V_SHE_ in (b)). The orbital images of the CB edge (orange isosurfaces) of (g) RICE-1 (potential range: −1.06 V_SHE_ to −1.37 V_SHE_ in (a)) and (h) RICE-2 (potential range: −1.05 V_SHE_ to −1.39 V_SHE_ in (b)). An isosurface level of 0.005 e Å^−3^ was used to generate the orbital images. C, N, and H are represented as brown, blue, and white spheres, respectively.

We used DFT and UV-vis absorbance measurements to probe the band structure of the COFs and evaluate their potential as visible light PCs. Through UV-vis absorbance measurements and applying the Kubelka–Munk formula (Fig. S12†), we estimated the band gaps to be 1.79 eV for RICE-1 and 1.75 eV for RICE-2, which corresponds to photons with 693 nm and 708 nm wavelengths, respectively. This suggests that the COFs could serve as PCs under visible light irradiation. In addition, we applied DFT to compute the bandgap center (BGC) position of RICE-1 and RICE-2 (Fig. 2a and b). We calculated the conduction band minimum (CBM) and valence band maximum (VBM) (Fig. 2c and d) for RICE-1 and RICE-2 from the experimental bandgap and the theoretical BGC (see ESI† eqn (S1) and (S2)). The CBM of the COFs (−1.43 V_SHE_ and −1.46 V_SHE_ for RICE-1 and RICE-2, respectively) are more negative than the redox potential of typical chain transfer agents (CTAs), such as 2-(*n*-butyltrithiocarbonate) propionic acid (BTPA) whose redox potential is −0.36 V_SHE_,^23^ suggesting electron transfer from a photo-excited COF to CTA is thermodynamically favorable (Fig. 2c and d). Simulations of the molecular orbitals (MO) at the edge of the valence band (VB) and conduction bands (CB) in COFs are shown in Fig. 2e–h. For both RICE-1 and RICE-2, we found MOs at the VB edge that are localized at the fragment related to the original TTPA monomers (Fig. 2e and f), while the orbitals at the CB edge are delocalized (Fig. 2g and h). This indicates that when the COFs are illuminated, the excited electrons at the CB edge are highly mobile. Furthermore, the localization of the orbitals at the VB edge is beneficial for charge carrier separation, as this prevents recombination of photo-excited electrons and photo-generated holes.^41^

**Fig. 3:**
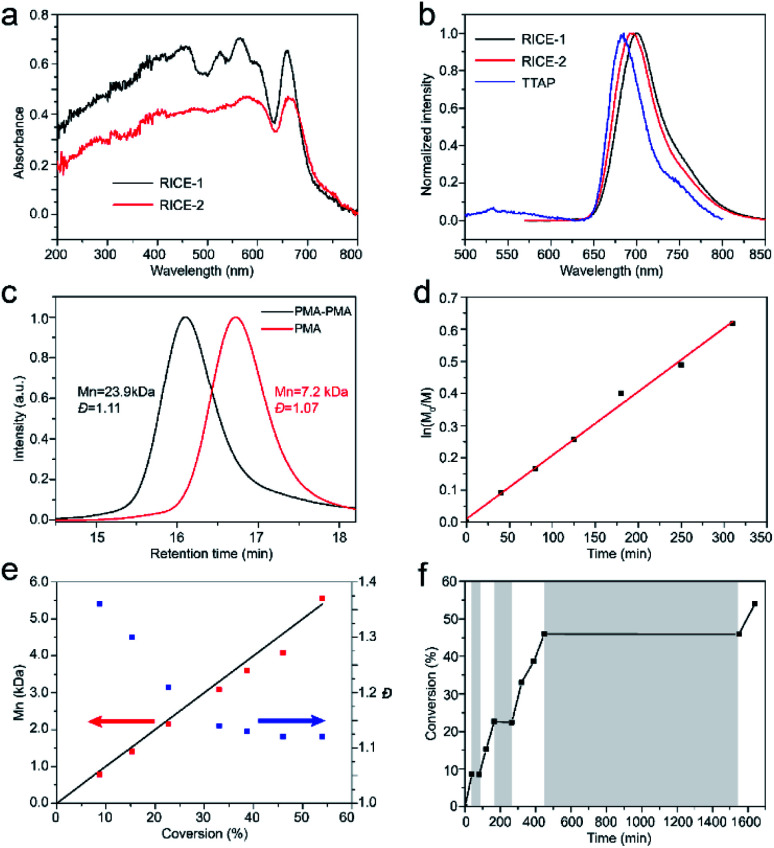
(a). Solid-state UV-vis spectra of the RICE COFs (b). Fluorescence emission spectra of RICE COFs and TTAP monomer in DMF. Excitation wavelength 460 nm. (c). GPC profiles of PMA and the diblock copolymer (d) first order kinetic plot of the polymerization of MA (DP = 100) (e) *M*_n_ and *Đ* of resulting polymers *versus* monomer conversion. Black line is theoretical *M*_n_*vs.* conversion. Blue dots are *Đ* at different conversion. Red dots are *M*_n_ at different conversion. (f) Pulsed-irradiation kinetic experiments. The shaded areas indicate the reaction operated in the dark while the unshaded areas represent the reaction under irradiation.

We investigated the optical properties of the two COFs through diffuse reflectance UV-vis spectroscopy (Fig. 3a). The absorption spectra of both COFs span from the UV to visible regions, with absorption edges at about 720 nm. The absorption edges of RICE-1 and RICE-2 are red shifted by about 55 nm compared to the Q band with lowest energy of porphyrin monomer (Fig. S13 and S14†) due to the extended conjugation in the COF frameworks. The fluorescence emission peaks of RICE-1 and RICE-2 were located at 701 nm and 692 nm (Fig. 3b). These are also red-shifted by 28 and 19 nm, respectively, compared to the TTAP. These optical properties suggest that both COFs could function as visible light PCs.

**Scheme 2:**
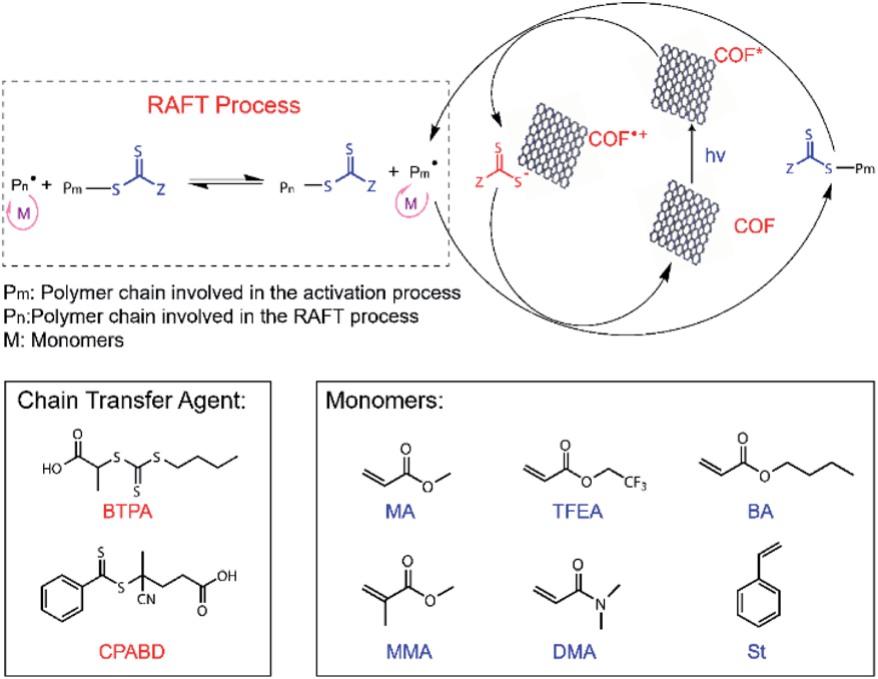
Proposed PET-RAFT polymerization mechanism using RICE COFs as PC and molecular structures of chain transfer agents (CTAs) and monomers used for PET-RAFT studies.

The excellent COF crystallinity, high surface area, broad absorption spectra, highly reductive CB, and efficient charge separation originating from the donor–acceptor structures of RICE COFs suggested that the RICE COFs were promising candidates for PCs for PET-RAFT polymerizations (Scheme 2). We first investigated the PET-RAFT polymerization of methyl acrylate (MA) using 1 mg ml^−1^ COF as the PC with a target degree of polymerization (DP) of 190. Polymerization was conducted in a polar solvent (*N*,*N*-dimethylformamide, DMF) to encourage electron transfer. Using RICE-1 as the PC under blue-light LED irradiation (15 mW cm^−2^), we achieved a substantial monomer conversion of 85.0% (Table 1, entry 1). Moreover, the product had a low molecular weight dispersity *Đ* of 1.06, and there was good agreement between the number-average molecular weight (*M*_n_) measured by gel permeation chromatography (GPC, *M*_n,GPC_ = 13.9 kDa) and the theoretical value (*M*_n,th_ = 14.2 kDa). Similarly, PET-RAFT polymerization using RICE-2 yielded monomer conversion of 79.6% (Table 1, entry 2) while maintaining excellent control (*Đ* = 1.05). Control experiments in the absence COF, light, or CTAs did not produce any polymer or consume monomer (Table S1,† entries 1–5), demonstrating the importance of COFs to these polymerization reactions. Additionally, using TTAP as a homogeneous PC produced only a trace amount of polymer (Table S1,† entry 6).

**Table 1:** Results from PET-RAFT polymerizations using RICE COFs^a^

Monomer	Monomer	Solvent	Light sources	CTA	Time (h)	Conv.^b^ (%)	*M*_n,GPC_ c (kDa)	*M*_n,th_ d (kDa)	*Đ* d
1^e^	MA	DMF	Blue	BTPA	17	85.0	13.9	14.2	1.06
2	MA	DMF	Blue	BTPA	17	79.6	13.4	13.3	1.05
3	MA	DMF	Green	BTPA	24	61.8	10.9	10.4	1.10
4	MA	DMF	Red	BTPA	36	50.1	8.6	8.4	1.10
5	MA	DMAC	Blue	BTPA	17	91.3	15.7	15.2	1.07
6	MA	MeCN	Blue	BTPA	17	82.1	14.9	14.4	1.09
7	MA	Toluene	Blue	BTPA	17	61.5	10.9	10.3	1.13
8	DMA	H_2_O	Blue	BTPA	12	92.1	19.5	17.6	1.14
9	BA	DMF	Blue	BTPA	14	74.0	16.1	18.2	1.07
10	TFEA	DMF	Blue	BTPA	14	88.9	27.0	26.3	1.06
11	MMA	DMF	Blue	CPADB	17	44.9	11.1	8.8	1.11
12	St	DMF	Blue	CPADB	29	32.9	9.3	6.8	1.09

a General conditions: target DP = 190. COF loading = 1 mg ml^−1^. Light intensity = 15 mW cm^−2^. RICE-2 was used as PC except for entry 1.

b Conversion was measured by ^1^H NMR.

c *M*_n_ measured by GPC in tetrahydrofuran, based on linear polystyrene as the calibration standard.

d *M*_n,th_ = DP × conversion × MW (monomer) + MW (initiator).

e RICE-1 was used as PC.

Next, we tested the performance of the RICE COFs under long wavelength (green and red) visible light. PCs that can function at these wavelengths are attractive because they require only lower-energy illumination and avoid potential side-reactions that occur under higher-energy UV and visible light.^42^ Polymerizations were successful under both green (*λ*_max_ ≈ 535 nm) and red (*λ*_max_ ≈ 635 nm) light, with monomer conversions of 61.8 and 50.1%, respectively, and a low molecular weight dispersity (*Đ* = 1.10) in the final products (see Table 1, entries 3–4). The apparent propagation rate (*k*^app^_p_) decreases with increased wavelength (Fig. S15†). These results demonstrate that the RICE COFs can be used as energy-efficient PCs active under green or red light illumination.

We further explored the generality and versatility of COF photocatalyzed PET-RAFT polymerization by conducting polymerization under different solvents and expanding the scope of monomers. Polymerization in two polar solvents *N*,*N*-dimethylacrylamide (DMAc) and acetonitrile (MeCN) were performed successfully, reaching high monomer conversions of 91.3% and 82.1%, respectively (Table 1, entries 5–6). Polymerization in a nonpolar solvent, toluene, resulted in a lower conversion of 61.5% for the same reaction time while still maintaining a low *Đ* of 1.13 (Table 1, entry 7). Moreover, RICE-2 COF successfully photocatalyzed PET-RAFT polymerization in water. Aqueous phase polymerization is always of general interest as it provides both environmentally friendly reaction conditions and access to functional hydrophilic polymers, which could be useful for specific bio-applications.^43–45^ The polymerization of water-soluble monomer *N*,*N*-dimethylacrylamide (DMA) was rapid, giving rise to 92.1% monomer conversion in 12 hours (Table 1, entry 8). An increased *k*^app^_p_ of the polymerization of DMA was also observed with the increased dielectric constant of the solvents (Fig. S16 and S17†). More importantly, we observed a high level of functional group tolerance for the PET-RAFT polymerization catalyzed by COF. The polymerization of butyl acrylate (BA) and 2,2,2-trifluoroethyl acrylate (TFEA) were successful, and we achieved considerable monomer conversions of 74.0% and 88.9%, respectively (Table 1, entries 9–10), after 14 hours of irradiation without sacrificing control over the polymerization reaction. Furthermore, we achieved 44.8% monomer conversion for the polymerization of methyl methacrylate (MMA) with a slightly higher *Đ* of 1.11 compared to acrylates (Table 1, entry 11). We found that the polymerization of styrene (St) was relatively slow and had only 32.9% conversion after 29 hours of reaction time (Table 1, entry 12). We attribute this to the lower reactivity of styrene compared to acrylates.^46^

Strong evidence for a living polymerization mechanism was obtained through chain extension studies and kinetic analysis of chain growth. As shown in Fig. 3c, we successfully chain extended poly(methyl acrylate) (PMA) using a macroinitiator with *M*_n_ of 7.2 kDa to produce a final polymer with *M*_n_ of 23.9 kDa and a low *Đ* of 1.11, indicative of living characteristics. Furthermore, as shown in Fig. 3d and e the polymerization followed first-order kinetics as evidenced by the linear relationship of ln([M]_0_/[M]) *versus* the irradiation time, the linear increase in *M*_n_ with monomer conversion, and decreased *Đ* with increased monomer conversion. A high degree of temporal control of the polymerization was illustrated by light “on” and “off” experiments (Fig. 3f), with no polymerization even for a dark period as long as 1100 minutes. Based on these results, we propose a PET-RAFT polymerization mechanism *via* the oxidation quenching cycle (Scheme 2). Briefly, after irradiation, photoexcited COF electrons reduce dormant RAFT chain transfer agents (CTAs), giving rise to radical species which can initiate or propagate chain growth, participate in the chain transfer process, or deactivate through reaction with the oxidized COF radical cations to form dominant state polymers and the ground-state COFs.

An important advantage of heterogeneous catalysts such as COFs over small molecular catalysts is that they can be easily recycled and reused. In our studies, RICE-2 was easily separated from the reaction mixture by centrifugation. The UV-vis of the reaction mixture after removing COFs showed negligible absorption or scattering peaks attributed to COF (Fig. S18†). Furthermore, PXRD and FT-IR showed that recycled COF maintained its crystallinity and structural integrity after reaction (Fig. S19 and S20†). The COF could be reused for PET-RAFT polymerization of MA at least 5 times, reaching stable monomer conversions (around 80%) and small *Đ* < 1.15 (Fig. S21 and Table S2†). The recyclability is also comparable to previously reported efficient PC, MOF-525 (Zn) (Table S3†).^30^

## Conclusions

In conclusion, we developed two porphyrin-based donor–acceptor COFs as PCs with a broad spectrum absorption. These COFs had exceptional photocatalytic performance in the PET-RAFT polymerization of various monomers under different solvents and irradiation conditions, resulting in high monomer conversions, excellent control over MW, and narrow molecular weight dispersities. The COF photocatalyzed RAFT polymerization exhibited features of a living polymerization reaction, including excellent temporal control over the polymerization, high chain-end fidelity of products, and a linear growth in polymer molecular weight with monomer conversion. This study demonstrates how COF photocatalysts can be rationally designed through proper choice of building blocks and provides opportunities for leveraging COFs as photocatalysts for a wide range of applications.

## Data availability

The datasets supporting this article have been uploaded as part of the ESI.†

## Author contributions

Y. Z., D. Z. and R. V. designed and conceptualized the research; Y. Z., D. Z., Q. Y., K. L., Y. L. and D. L. performed research; Y. C., C. Y. L. and T. P. S. performed the DFT analysis; all authors analyzed the data and discussed the results; Y. Z., D. Z., Y. C., C. Y. L., X. W., T. P. S. and R. V. wrote and revised the paper. R. V supervised the whole project.

## Conflicts of interest

There are no conflicts to declare.

## Supplementary Material

SC-012-D1SC05379E-s001
