# Supplementary material for: Porphyrin-based donor–acceptor COFs as efficient and reusable photocatalysts for PET-RAFT polymerization under broad spectrum excitation
Source: Chem Sci. 2021 Nov 26;12(48):16092–9. doi: 10.1039/d1sc05379e (PMC8672717; doi:10.1039/d1sc05379e)
Supplement: SC-012-D1SC05379E-s001 [file SC-012-D1SC05379E-s001.pdf]

Electronic Supplementary Material (ESI) for Chemical Science.
This journal is © The Royal Society of Chemistry 2021

Supporting Information

Porphyrin-based donor-acceptor COFs as efficient and reusable photocatalysts for PET-RAFT polymerization under broad spectrum excitation

Yifan Zhu,[+a] Dongyang Zhu,[+b] Yu Chen,[+b] Qianqian Yan,[a] Chun-Yen Liu,[b] Kexin Ling,[c] Yifeng Liu,[a] Dongjoo Lee, [b] Xiaowei Wu,[d, e*] Thomas P. Senftle,[b*] Rafael Verduzco[*a,b]

a. Department of Materials Science and Nanoengineering, Rice University, Houston, Texas, 77005, United States

b. Department of Chemical and Biomolecular Engineering,Rice University, Houston, Texas, 77005, United States, email: tsenftle@rice.edu, rafaelv@rice.edu

c. Department of Chemistry, Rice University, Houston, Texas, 77005, United States

d. CAS Key Laboratory of Design and Assembly of Functional Nanostructures, Fujian Provincial Key Laboratory of Nanomaterials, Fujian Institute of Research on the Structure of Matter (FJIRSM), Chinese Academy of Sciences, Fuzhou 350002, China, email: xmwuxiaowei@fjirsm.ac.cn

e. Xiamen Key Laboratory of Rare Earth Photoelectric Functional Materials, Xiamen Institute of Rare Earth Materials (XMIREM), Haixi Institutes, Chinese Academy of Sciences, Xiamen 361021, China

+ These authors contributed equally to this work

Materials and Methods

All chemicals were purchased from commercial sources and used without further purification unless specific treatment mentioned. 5,10,15,20-Tetrakis(4-aminophenyl)porphyrin (TTAP) , 4',4'',4''',4''''-(ethene-1,1,2,2-tetrayl)tetrabiphenyl-4-aldehyde (ETTA) and 4,4',4'',4'''-([1,1'-Biphenyl]-4,4'-diylbis(azanetriyl))tetrabenzaldehyde (BDTA) were purchased from YanShen Technology Co. Hexanes (anhydrous grade, 95%), toluene (anhydrous, 99.8%), acetone (99.8%), tetrahydrofuran (THF, inhibitor-free, for HPLC, ≥99.9%), were purchased from Fisher Scientific. Benzyl alcohol (anhydrous, 99.8%, BzOH), mesitylene (98%), dimethylformamide (anhydrous, 99.8%, DMF) acetonitrile (anhydrous, 99.8%, MeCN), methyl methacrylate (MMA), butyl acrylate (BA), methyl acrylate (MA), 2,2,2-Trifluoroethyl acrylate (TFEA), 4-cyano-4-(phenylcarbonothioylthio)pentanoic acid (CPADB) and styrene(St) were purchased from Sigma-Aldrich. All monomers were used after disinhibition by percolating over a column of the mixture of neutral alumina and silica gel (particle size 0.063 nm-0.200 nm). 2-(n-butyltrithiocarbonate)-propionic acid (BTPA) were synthesized according to literature procedures.

General instrumentation

Nuclear Magnetic Resonance (NMR) spectra was obtained on an NMR Bruker 500 MHZ operated at room temperature. Absorption spectra were measured with Agilent Cary60 UV-Vis spectrometer. Photoluminescence spectra were carried out on Cary Eclipse

fluorometer. Gel permeation chromatography (GPC) was carried out on the Agilent 1260 Infinity LC system and calibrated with polystyrene (PS) standards in THF. The house hold LED (High led spot lamp, HK LD302, maximum power =10 W, light intensity was adjusted to 15 mW/cm^2) has a tunable intensity and wavelength from blue light to red light (λ_{max} of blue LED is 460 nm, green LED is 535 nm and red LED is 635 nm.). PXRD data were recorded on a Rigaku SmartLab XRD from 2θ = 1 up to 30° with a 0.02° increment. Powder samples were leveled flat on zero background sample holders. Fourier transform infrared spectra (FT-IR) of all solid samples were measured using a ThermoNicolet iS10 FT-IR spectrometer with a diamond ATR attachment and are uncorrected. Nitrogen sorption measurements were conducted on a Quantachrome Autosorb-iQ-MP/Kr BET Surface Analyzer. All samples were dried and degassed at 80 °C for 12 h and backfilled with N_2. BET surface areas were determined using BET adsorption models included in the instrument software (ASiQwin version 5.2). Scanning electron microscopy (SEM) was performed on an FEI Quanta 400 FESEM operating at 30.00 kV. Each sample was coated with a 10 nm gold film using a Denton Desk V Sputter. Thermo-gravimetric Analysis (TGA) was performed on Q-600 Simultaneous TGA/DSCX-ray photoelectron spectroscopy (XPS) was performed on a PHI Quantera XPS, which used a focused monochromatic Al Kα X-ray (1486.7 eV) source for excitation. The 50 W, 15 kV, and 200 μm diameter X-rays were shot on the sample. The XPS survey scan spectra in the 1100–0 eV binding energy range were recorded in 0.5 eV steps with a pass energy of 140 eV. High-resolution scan spectra were recorded in 0.1 eV steps with a pass energy of 26 eV. Low

energy electrons and Ar^+ ions were conducted for specimen neutralization in each measurement.

General procedure for the synthesis of RICE-1 and RICE-2

TTAP (0.01 mmol) and ETTA or BDTA (0.01 mmol) were weighed and dissolved in a mixture of 0.33 mL of benzyl alcohol and 0.67 mL of mesitylene in a Pyrex tube. Before the tube was sealed, 0.1 ml 6M acetic acid was added, and the solution was sonicated for 5 min. The sealed tubes were then transferred into an oven and heated at 120 °C for 7 days. All of the products were separated and washed thoroughly using THF, DMF and acetone. For vacuum drying, final solids were washed and immersed in hexane prior to drying. After filtration, samples were dried in vacuum oven at 80 °C overnight. The COFs were insoluble in water and all organic solvents tested, including acetonitrile (MeCN), hexanes, N,N-dimethylformamide (DMF), and N, N-dimethylacrylamide (DMAc).

General procedure for RICE COFs photocatalyzed PET-RAFT polymerization

To a 4 ml vial, 0.5 mg RICE COF, 0.25 ml solvent, 0.25 ml monomer and the corresponding amount of RAFT agents (BTPA or CPADB) were mixed together in the glove box. For Table 1, the degree of polymerization (DP) was 190. For the kinetic study (Figure 4d-f), DP was 100. The reaction vial was then sealed and sonicated for around 10 min until COF was well dispersed in the solution. The reaction vial was constantly stirred at ambient temperature under irradiation of a household LED lamp (λ_{max} = 460 nm for the

blue LED, λ_{max} = 535 nm for the green LED, λ_{max} = 635 nm for the red LED). The reaction vial was placed approx. 0.15 cm from the lamp, where the light intensity was 15 mW/cm^2, unless otherwise specified. The whole setup was covered with aluminum foil to block exposure to any other light sources. The monomer conversion was determined by ^1H NMR.

General procedure for first order kinetic study

To study the reaction kinetics, polymerization was carried out under 15 mW/cm^2 blue LED in the glove box. To a 4 ml vial, 1 mg RICE-2, 0.25 ml DMF, 0.25 ml MA and 6.55 mg BTPA (DP=100) were mixed together in the glove box. The reaction vial was then sealed and sonicated for around 10 min until COF was well dispersed in the solution. The mixture was constantly stirred at ambient temperature. At specific time points, 0.01 ml mixture was taken and dissolved in 0.7 ml deuterated chloroform for ^1H NMR. The NMR solution was further concentrated and dissolved in THF for GPC measurement.

General procedure for chain extension

Yielding PMA was precipitated in MeOH, filtered and redissolved in THF. This process was repeated two more times to make sure no residual initiators inside. The purified PMA (6 mg), momomer (0.15 mL), 1 mg RICE-2 and 0.25 ml DMF was deaerated by three freeze-pump-thaw cycles, backfilled with argon, and stirred at ambient temperature under irradiation of a household blue LED floodlight for 6 h.

General procedure for COF recycling

Polymerization mixtures were diluted with 2 ml THF and centrifuged at 5000 rpm. The supernatant was removed and 5 ml THF was added following by centrifugation. This procedure was repeated three times to ensure all the impurities were washed out. For vacuum drying, final solids were washed and immersed in hexane prior to drying. After filtration, samples were dried in a vacuum oven at 80 °C overnight. The PET-RAFT polymerization was conducted following general procedure. Briefly, to a 4 ml vial, 0.5 mg recycled RICE-2, 0.25 ml DMF, 0.25 ml MA and 3.45 mg BTPA (DP = 190) were mixed together in the glove box. The reaction vial was then sealed and sonicated for around 10 min until COF was well dispersed in the solution. The reaction vial was constantly stirred at ambient temperature under irradiation of a household LED lamp (λ_{max} = 460 nm).

Density functional theory simulations

A donor–acceptor (D-A) system is desirable for COFs with superior photocatalytic performance. In this work, the highest occupied molecular orbital (HOMO) and lowest unoccupied molecular orbital (LUMO) of the monomers, i.e., ETTA, BDTA, and TTAP, were computed by cluster calculations using density functional theory (DFT) in NorthWest Chemistry (NWChem) modeling software.[1] Becke, 3-parameter, Lee−Yang−Parr (B3LYP) exchange-correlation functional[2,3] with the Dunning aug-cc-pVDZ basis set[4,5] was used for geometry optimization of monomers and determining their HOMO and

LUMO energy level in vacuum. In addition, periodic DFT simulations were performed to obtain electronic properties of synthesized 2D COFs, i.e., RICE-1 and RICE-2, using the Vienna *ab initio* Software Package (VASP) 5.4.4.[6,7] We simulated the electronic structure of COFs using the Perdew–Burke–Ernzerhof (PBE) exchange-correlation functional,[8] and the plane-wave basis sets were truncated to a kinetic energy cutoff of 550 eV. The projector augmented wave (PAW)[9] method with default VASP potentials[10] was applied to treat core electrons. Valence electrons (C-$2s^2 2p^2$, N-$2s^2 2p^3$, and H-$1s^1$) were treated self-consistently. All calculations were spin-polarized. The Grimme DFT-D3 dispersion correction was applied to treat van der Waals interactions.[11] Gaussian smearing was employed with a smearing width of 0.05 eV, and self-consistent-field electronic energy was converged to 10^{-5} eV. Geometries were optimized to a convergence criterion of 0.02 eV $Å^{-1}$. The periodic structure of COFs and the unit cell parameters, a and b, were obtained from powder X-ray diffraction (PXRD) measurements and structural simulations, while the cell dimension c is determined from the unit cell with the minimum energy with respect to the interlayer distance of the COFs determined with a series of single-point calculations. Then we conducted cell relaxation to reduce Pulay stress in the plane-wave DFT simulations. The optimized lattice constants were (31.05 Å, 26.29 Å, 6.50 Å) and (26.06 Å, 26.06 Å, 5.91 Å) for RICE-1 and RICE-2, respectively. A Monkhorst-Pack[12] (MP) k-point mesh of $1\times1\times4$ was sampled on the periodic COF simulation models.

Note that band edge positions computed directly by Kohn-Sham (KS) DFT are well known to suffer errors related to the derivative discontinuity of the exchange-correlation

energy, i.e., the standard generalized gradient approximation (GGA) functionals, like PBE, underestimate the bandgap.[13,14] However, the BGC position computed with KS-DFT is formally exact for a wide range of semiconducting materials,[15] such as metal oxides, GaP, and SiC. Thus, we only used DFT calculations to determine the bandgap center (E_{BGC}). The valence band maximum (VBM) and the conduction band minimum (CBM) of the COFs were determined from the calculated E_{BGC} and the experimentally measured bandgap (E_g), using Eqns. S1-S2[16]:

$$E_{VBM} = E_{BGC} - ½ E_g \tag{S1}$$

$$E_{CBM} = E_{BGC} + ½ E_g \tag{S2}$$

In this work, the computed potentials are referenced to standard hydrogen electrode (SHE), i.e., 4.44 V (vs. vacuum potential).[17] The vacuum potential of the simulation cell is determined by the electrostatic potential at the center of the internal pore of the COFs.[18,19]

Supporting data

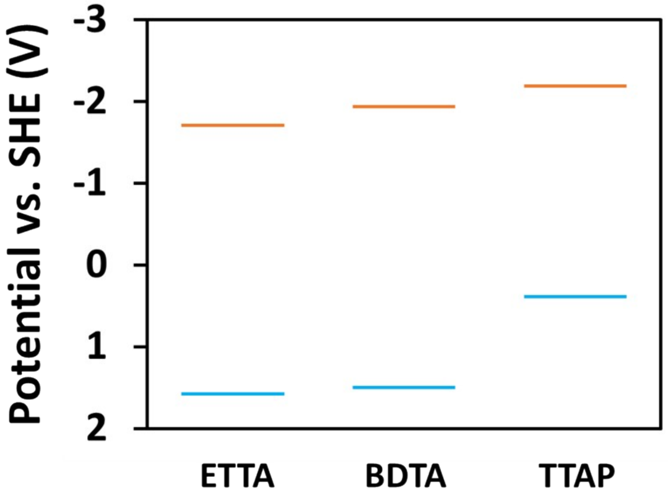

Figure S1. HOMO (blue solid lines) and LUMO (orange solid lines) energy levels of the monomers, i.e., ETTA, BDTA, and TTAP.

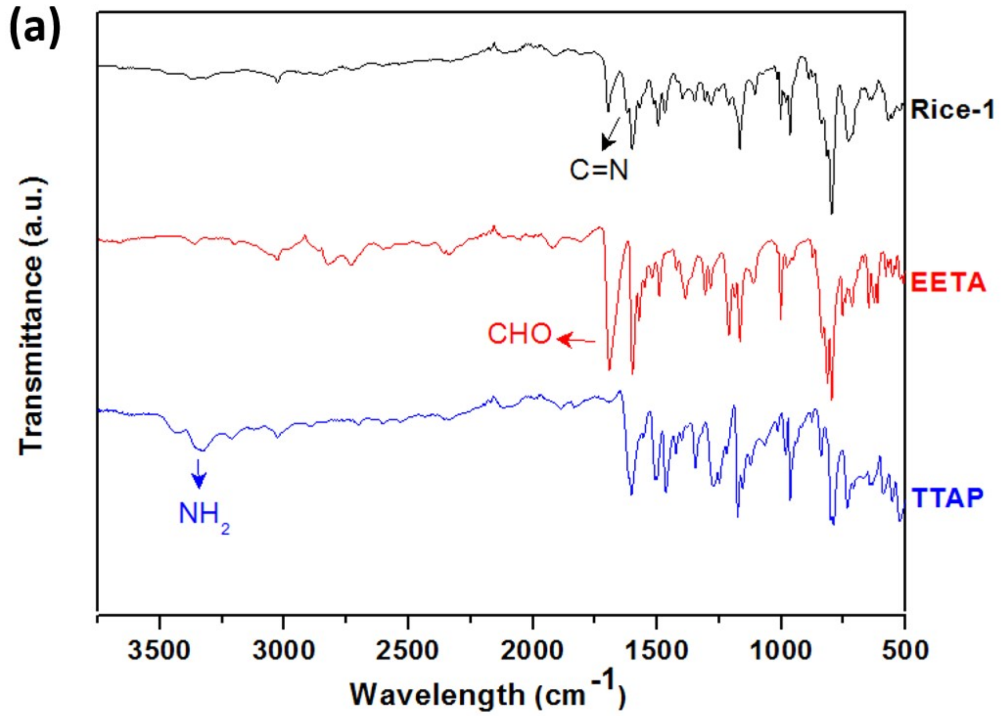

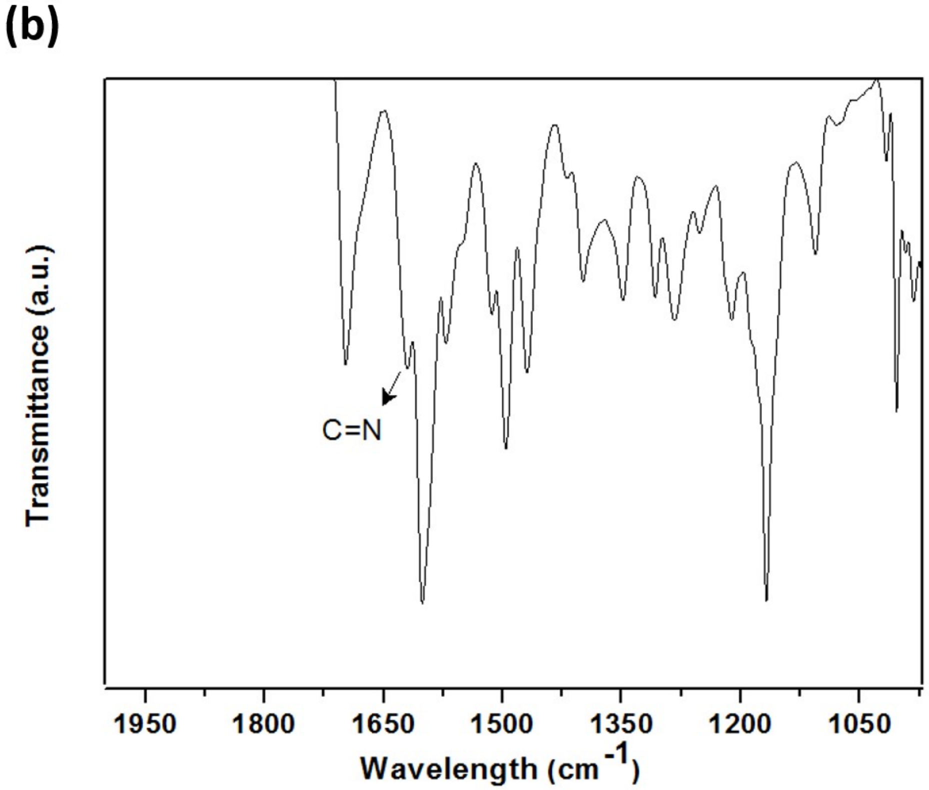

Figure S2. (a) FT-IR of RICE-1 (black line), TTAP monomer (blue line) and EETA monomer (red line). (b) Enlarged FTIR of RICE-1.

Note: Through Fourier transform infrared (FT-IR) spectroscopy, we verified the presence of C=N stretching bands at 1621 cm^{-1} for RICE-1 along with the disappearance of C=O and N-H stretching bands present for the starting materials

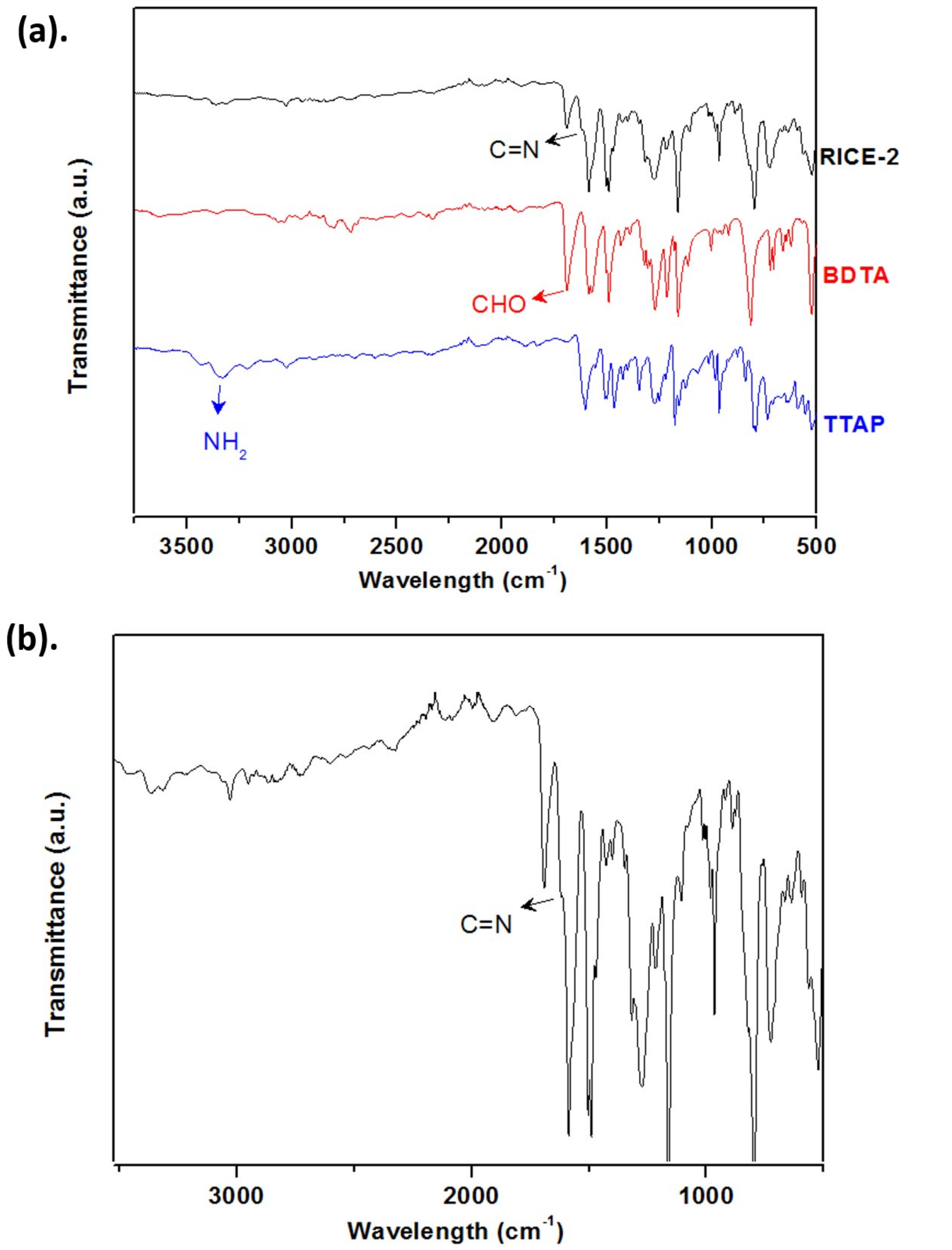

Figure S3. (a). FT-IR of RICE-2 (black line), TTAP monomer (blue line) and EETA monomer (red line). (b). Enlarged FT-IR of RICE-2.

Note: The presence of C=N stretching bands at 1617 cm^{-1} for RICE-2.

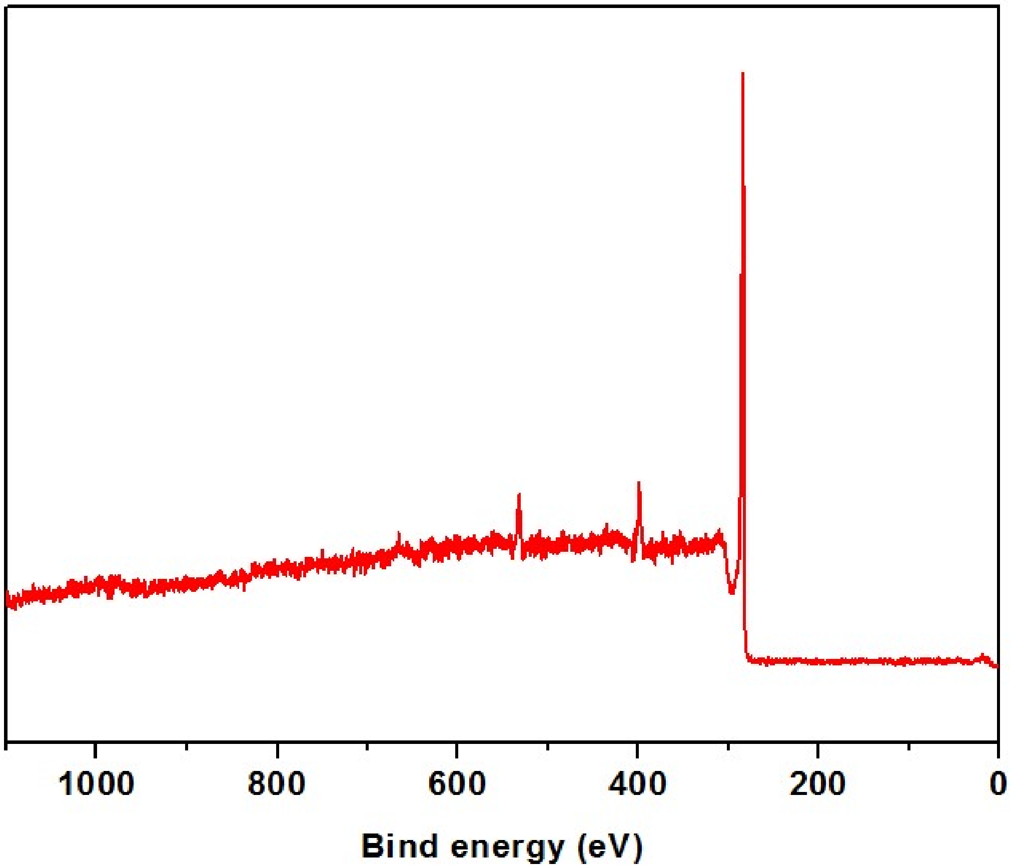

Figure S4. XPS survey scans of RICE-1

Note: X-ray photoelectron spectroscopy (XPS) survey scans exhibited peaks near 284, 397 and 532 eV, which we attributed to the C1s, N1s, and O1s binding energies, respectively

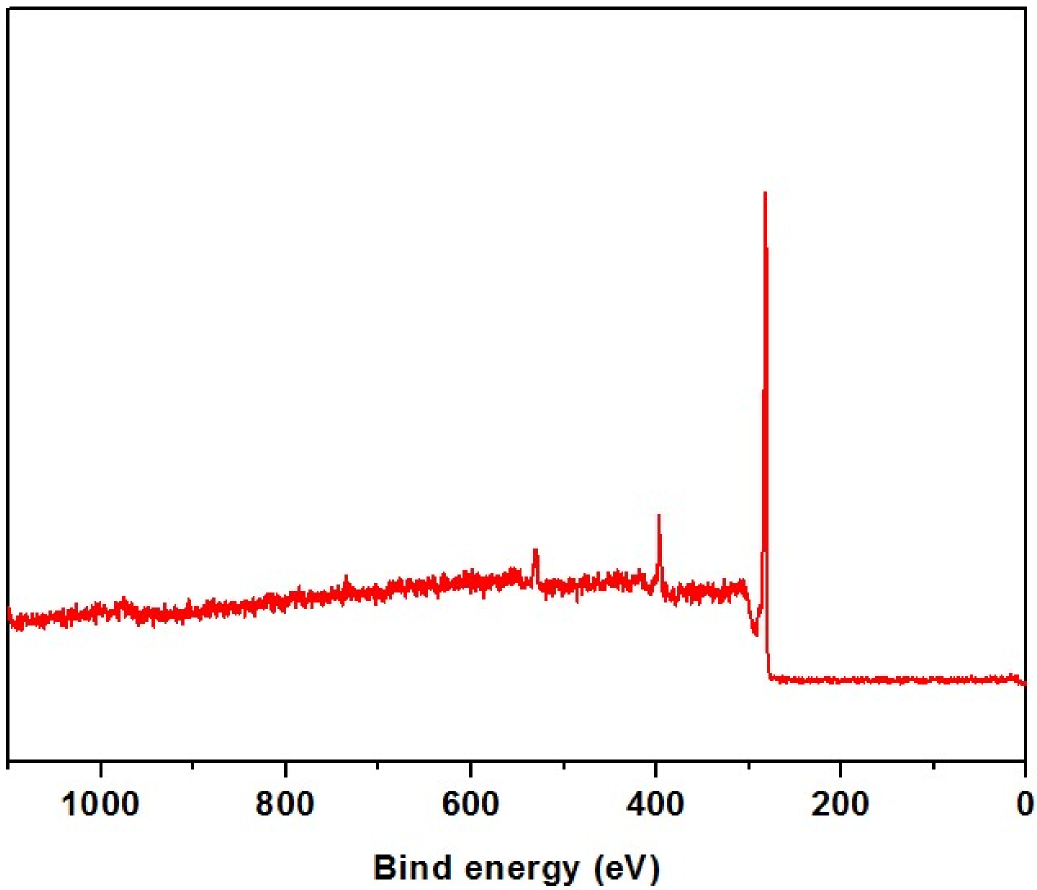

Figure S5. XPS survey scans of RICE-2.

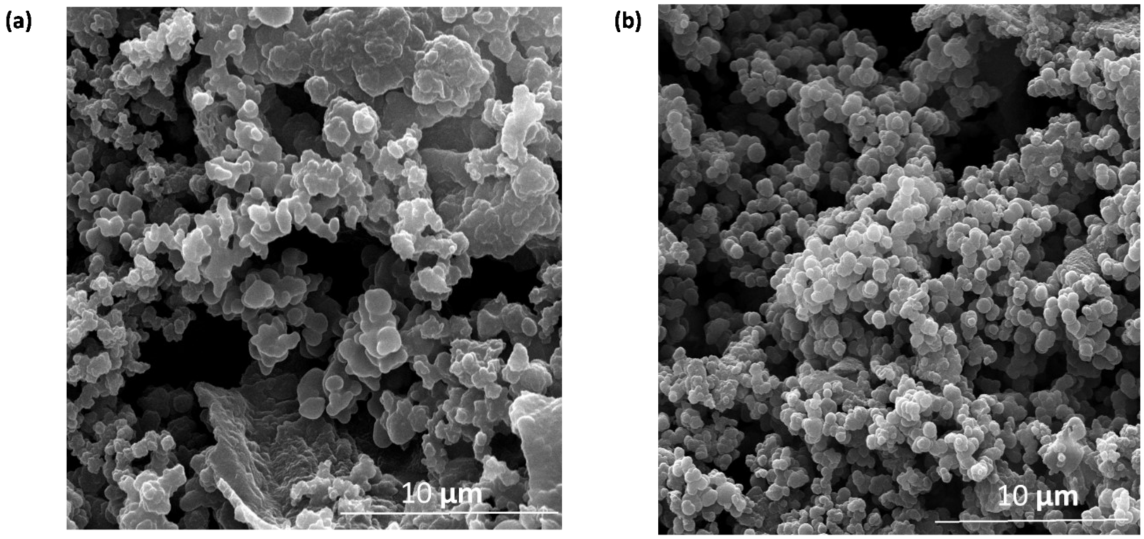

Figure S6. SEM of (a). RICE-1 and (b). RICE-2.

Note: Scanning electron microscopy (SEM) images of both COFs displayed aggregated macroparticles morphology

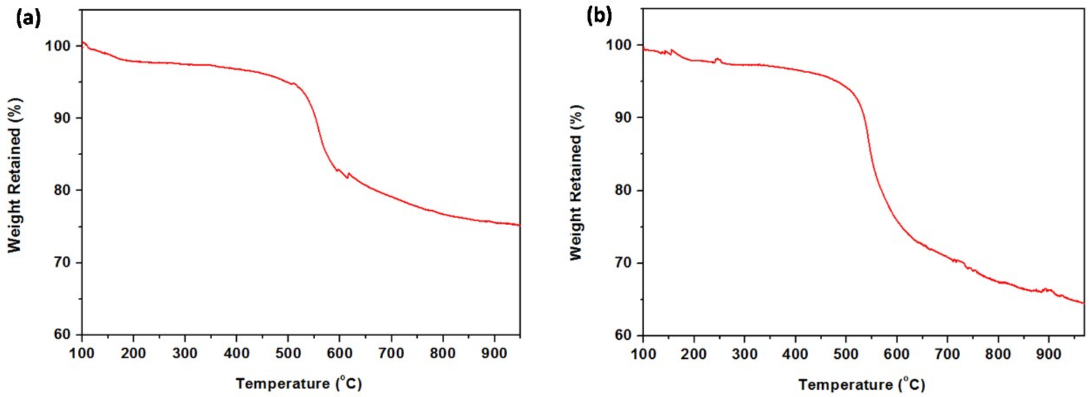

Figure S7. TGA of (a). RICE-1 and (b). RICE-2.

Note: Thermogravimetric analysis (TGA) revealed that both COFs are thermally stable up to 500 ℃, indicative of their excellent thermal stabilities.

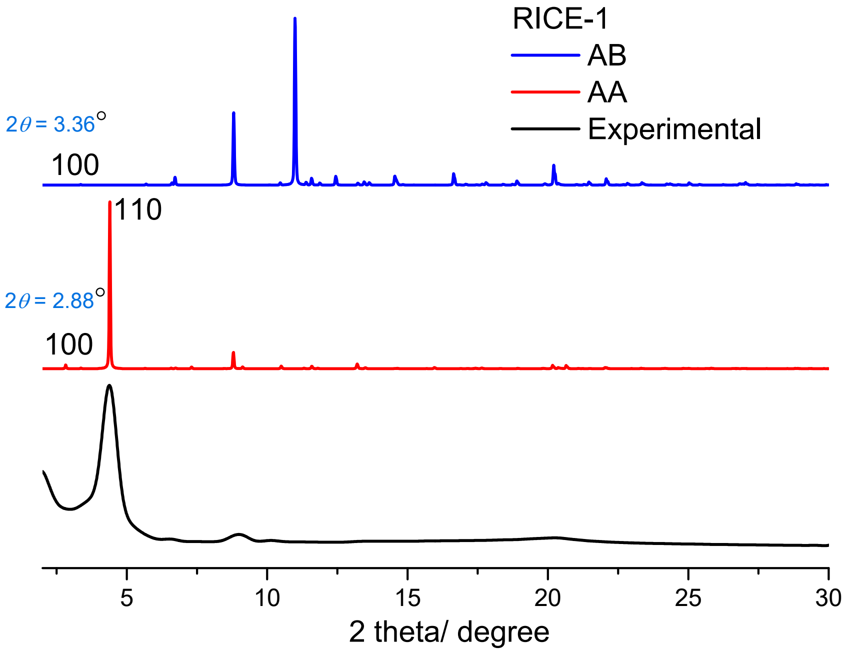

Figure S8. PXRD patterns of RICE-1 with the experimental profiles in black, simulated AB stacking model in red and simulated AB stacking model in blue.

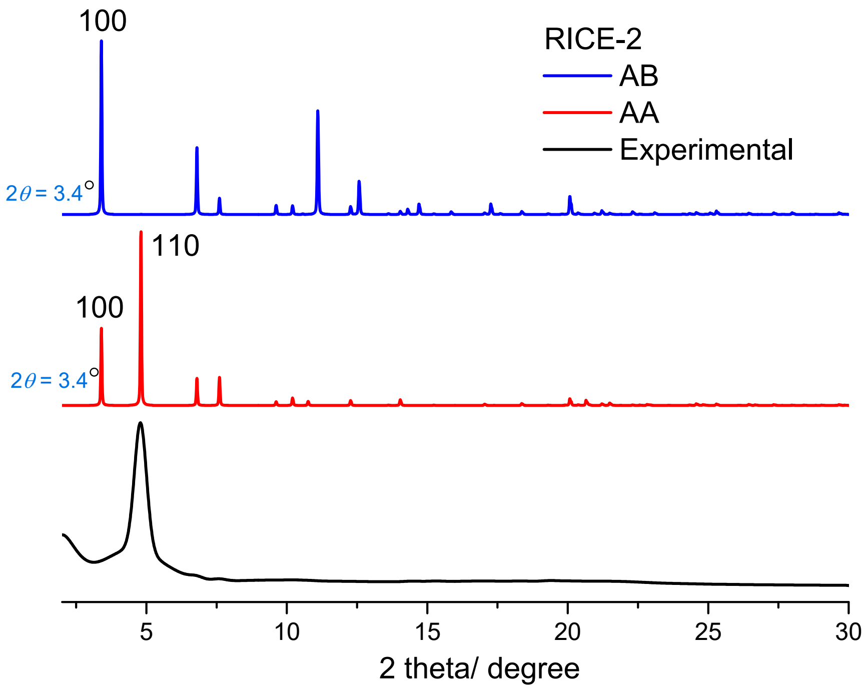

Figure S9. PXRD patterns of RICE-2 with the experimental profiles in black, simulated AA stacking model in red and simulated AB stacking model in blue.

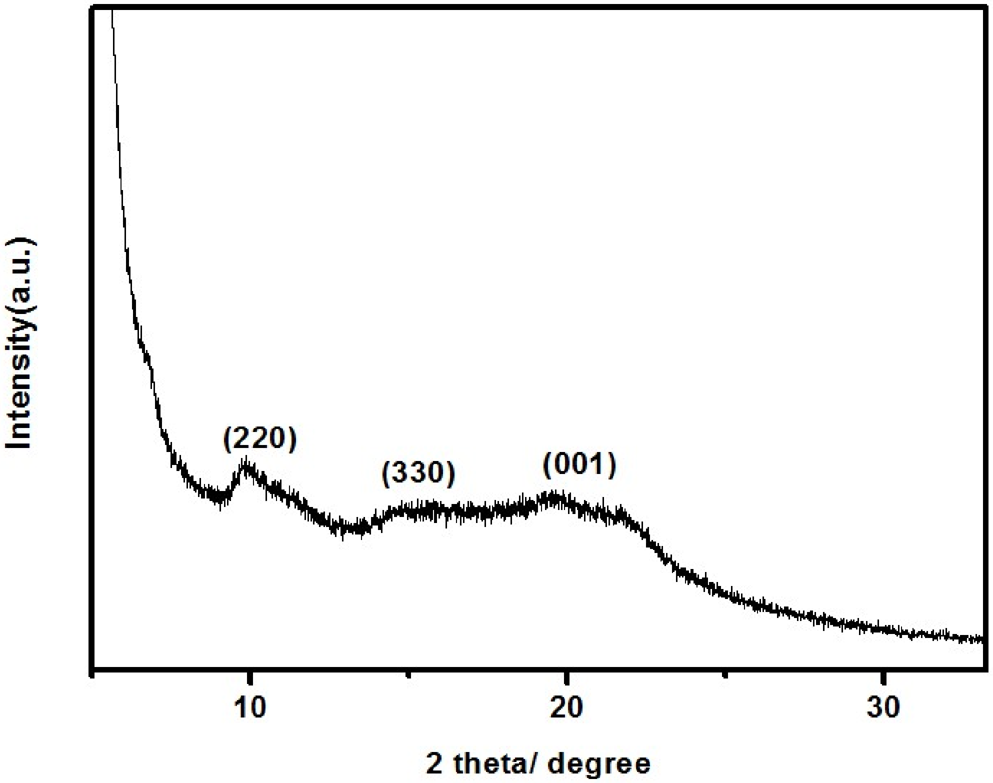

Figure S10. Enlarged PXRD pattern of RICE-2 with 2theta ranging from 6-32 degree.

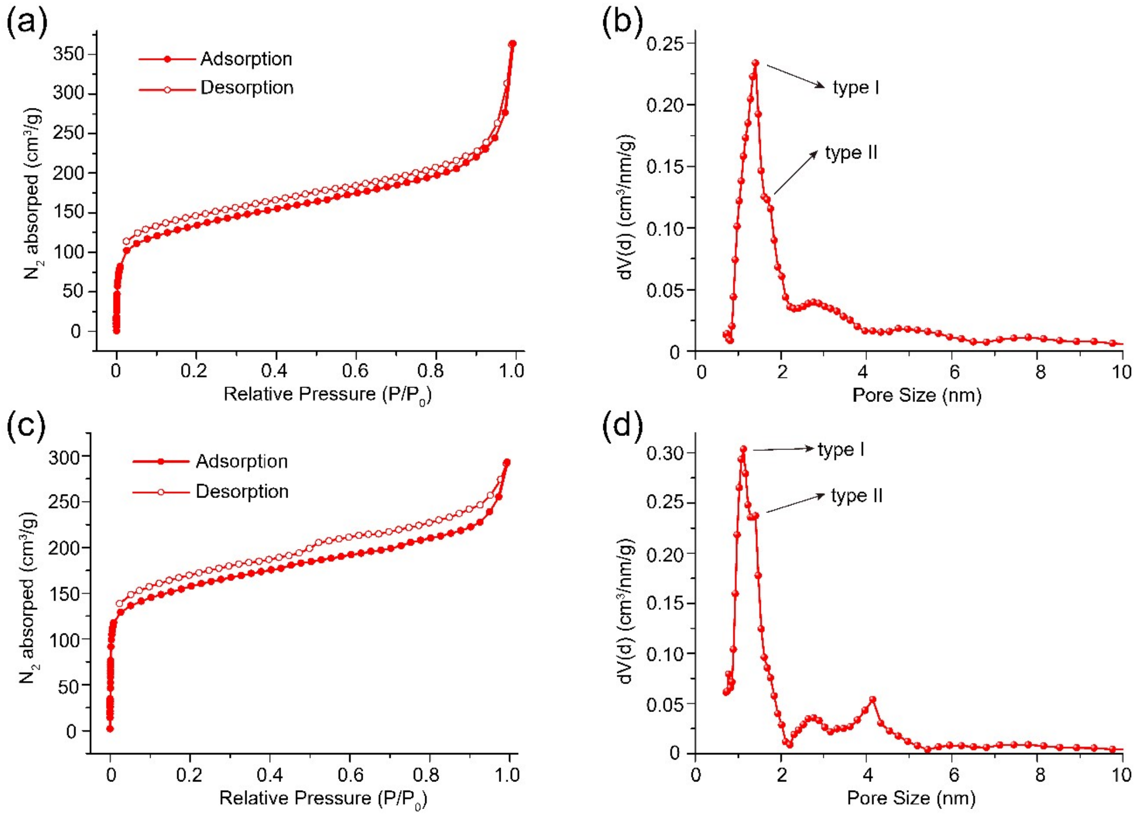

Figure 11. BET isotherms of (a) RICE-1 and (c) RICE-2. Pore size distribution of (b) RICE-1 and (d) RICE-2.

Note: From the simulation in Figure 2, RICE-1 and RICE-2 have two types of pores (labeled in Figures S11b and S11d).

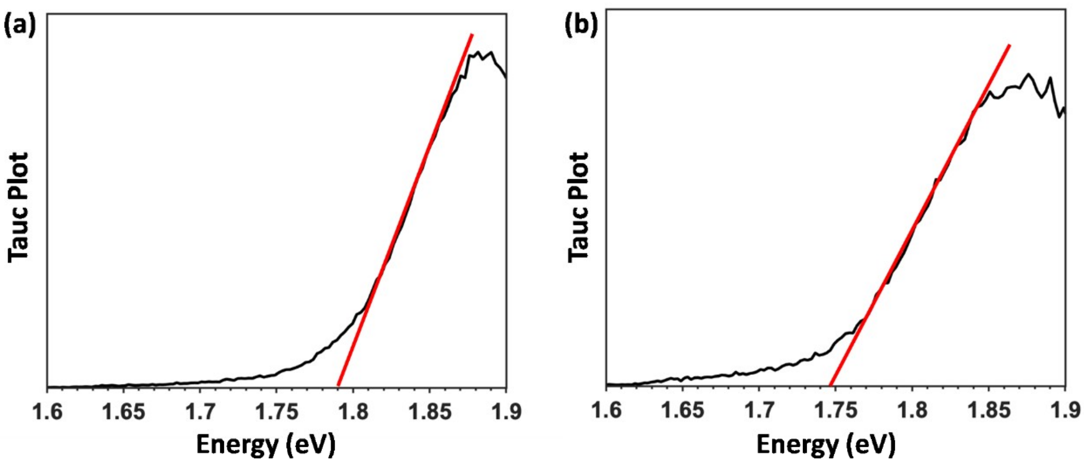

Figure S12. Tauc plot of absorption spectra obtained from the Kubelka–Munk function (Figure 3a) and the linear fit for direct band gaps of the COFs of (a). RICE-1 and (b). RICE-2.

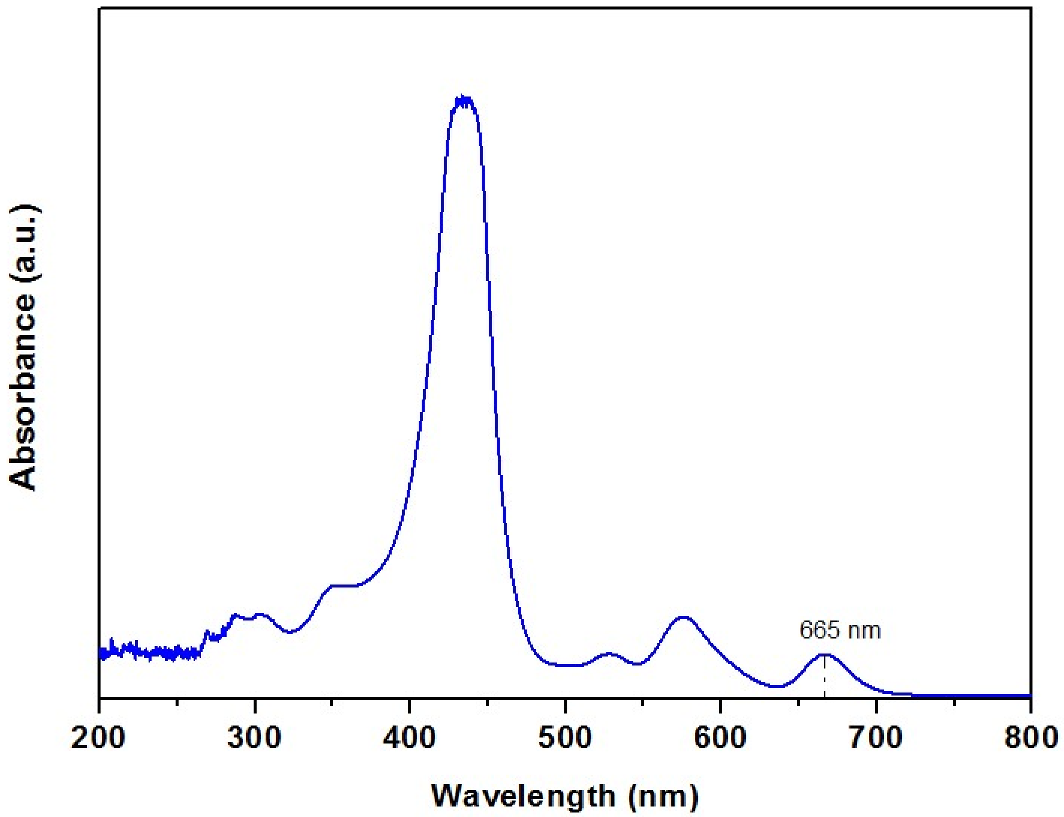

Figure S13. UV-vis absorption of TTAP monomer.

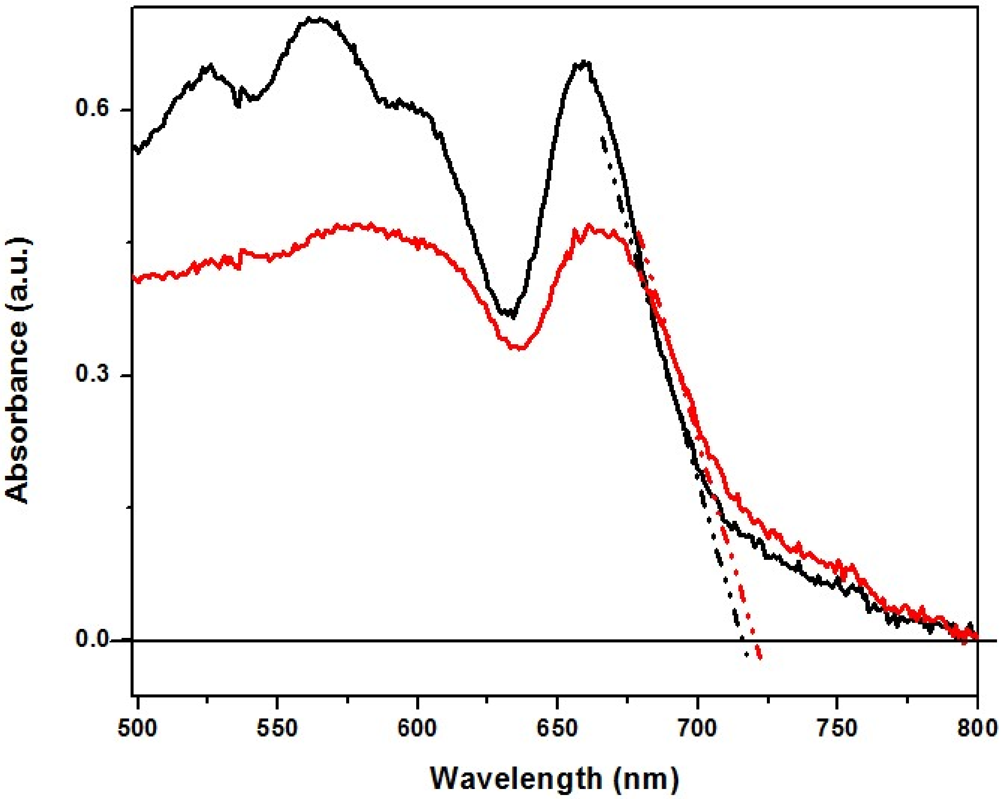

Figure S14. Enlarged picture of the absorption of RICE-1 (black) and RICE-2 (red) with marked absorption edge.

Note: Absorption edge of RICE-1 is 718 nm and of RICE-2 is 721 nm.

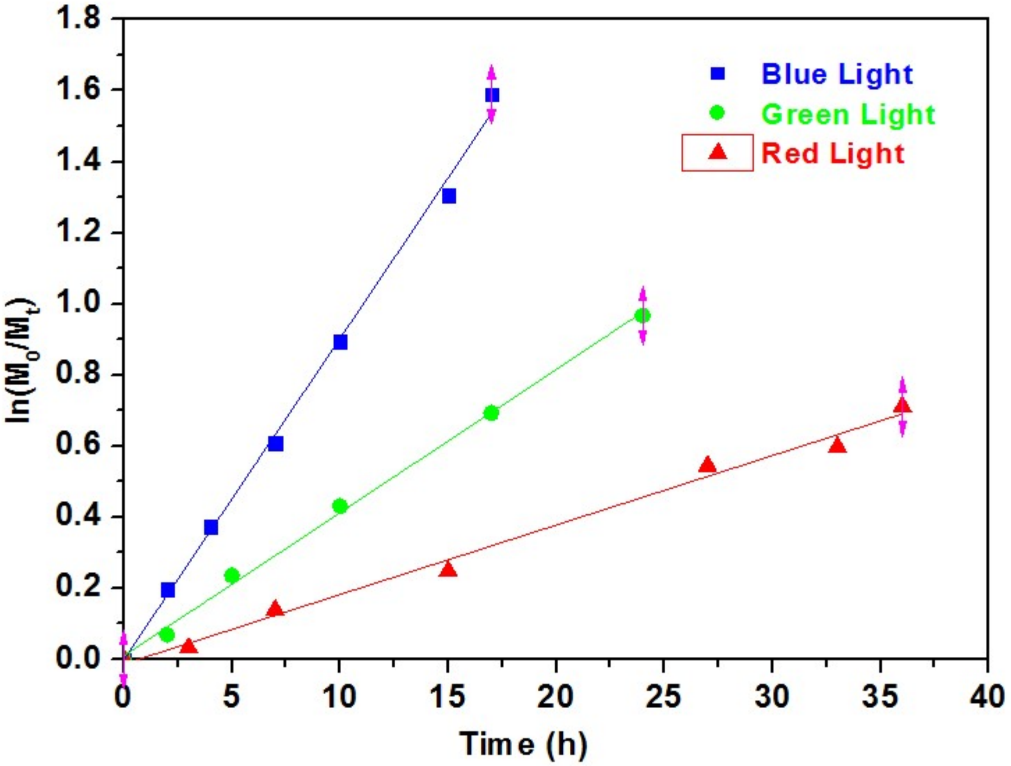

Figure S15. Kinetic study of PET-RAFT polymerization under different irradiation wavelength using the reaction conditions in Table 1, entries 2-4.

Notes: k_p^{app} (blue light) = 0.0015 min $^{-1}$; k_p^{app} (green light) = 0.00068 min $^{-1}$; k_p^{app} (red light) = 0.00033 min $^{-1}$.

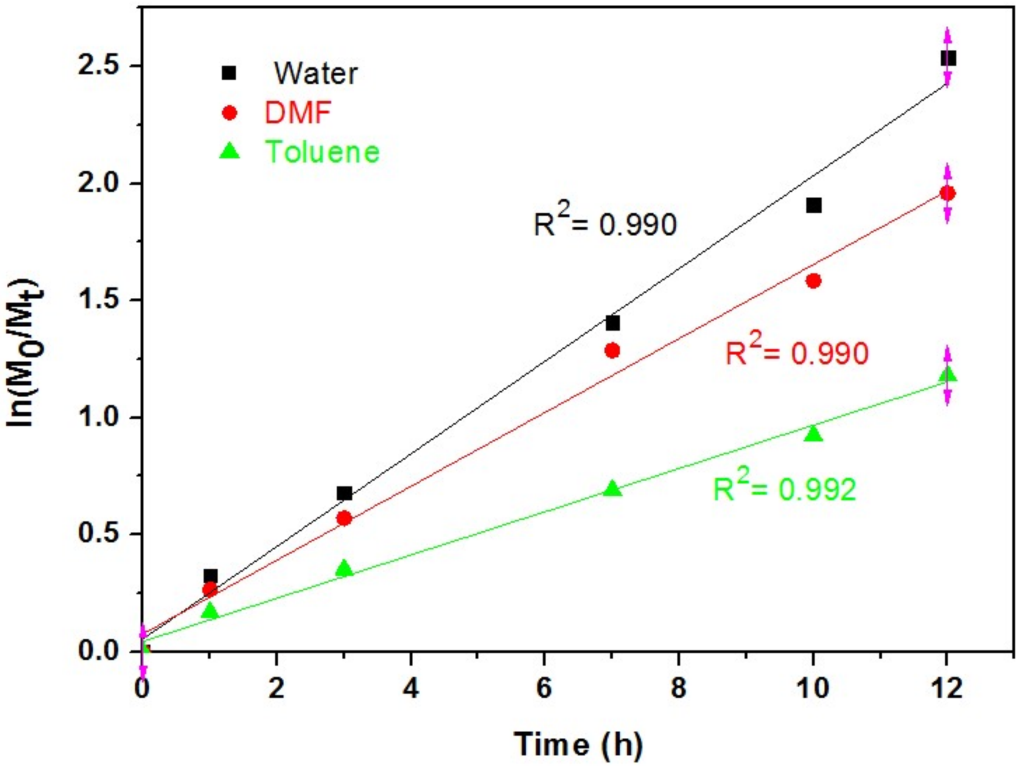

Figure S16. Kinetic study of PET-RAFT polymerization of DMA using RICE-2 in different solvents. Reaction conditions: DP= 190, COF loading = 1 mg/ml. Light intensity = 15 mW/cm^2.

Notes: k_p^{app} (Water) = 0.0033 min^{-1}; k_p^{app} (DMF) = 0.0026 min^{-1}; k_p^{app} (Toluene) = 0.00153 min^{-1}.

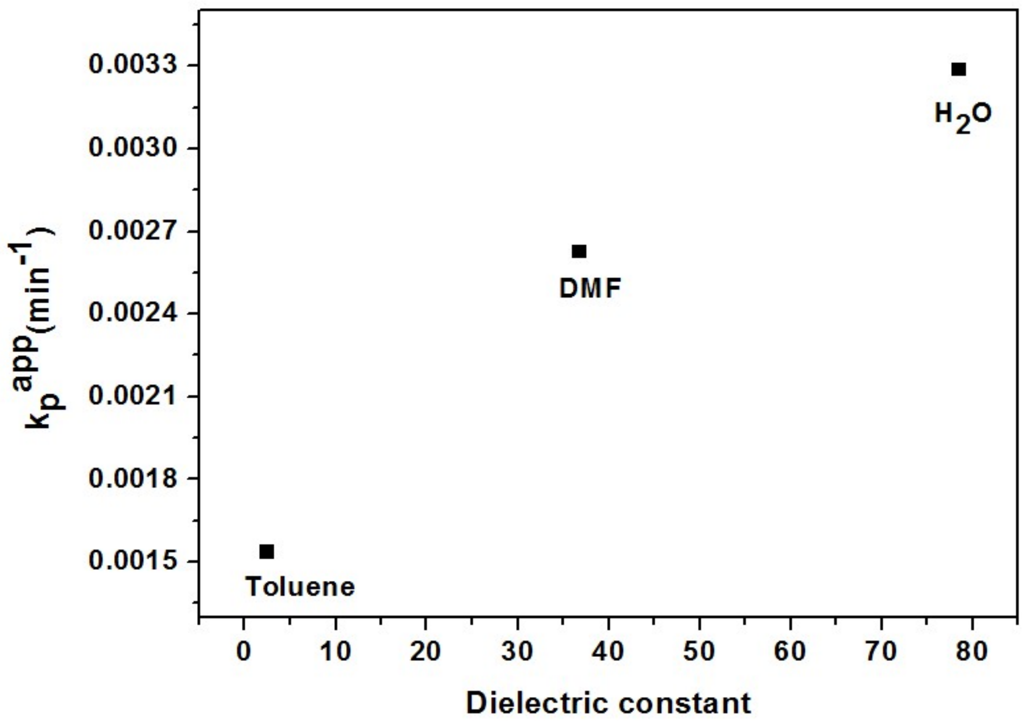

Figure S17. Apparent propagation rate (k_p^{app}) *vs*. dielectric constant. k_p^{app} was obtained from Figure 16.

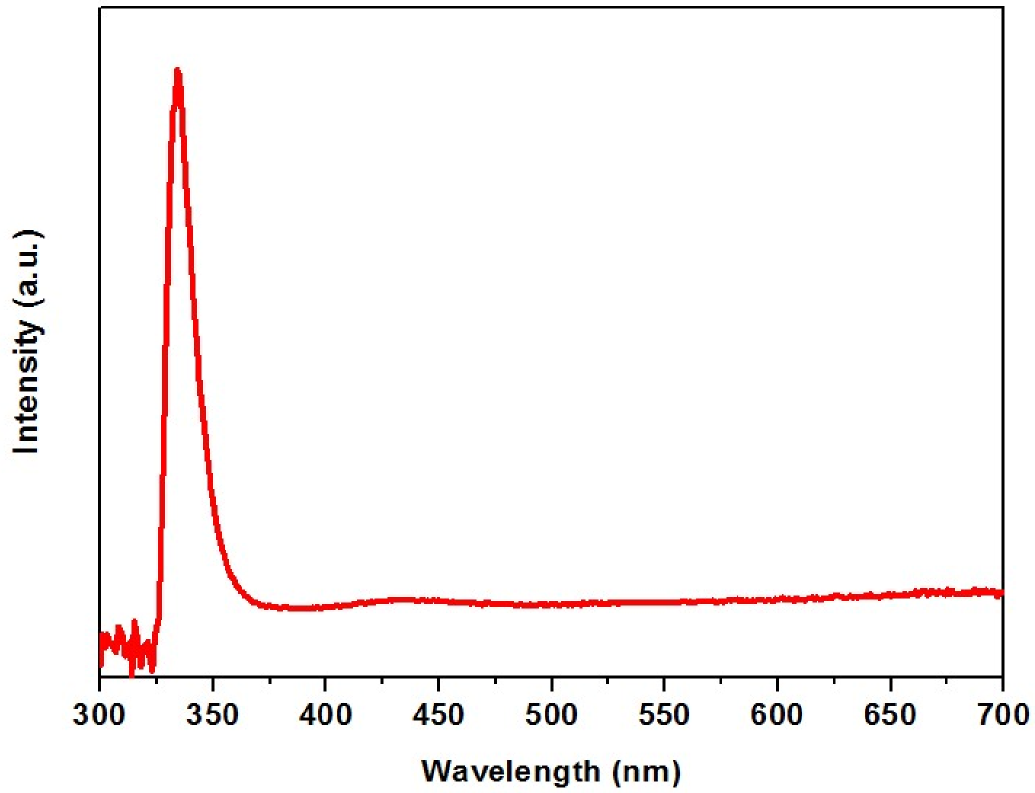

Figure 18. UV-vis of the polymerization mixture after centrifugation. The absorption spectra corresponding well with the absorption of the RAFT agent (BTPA).

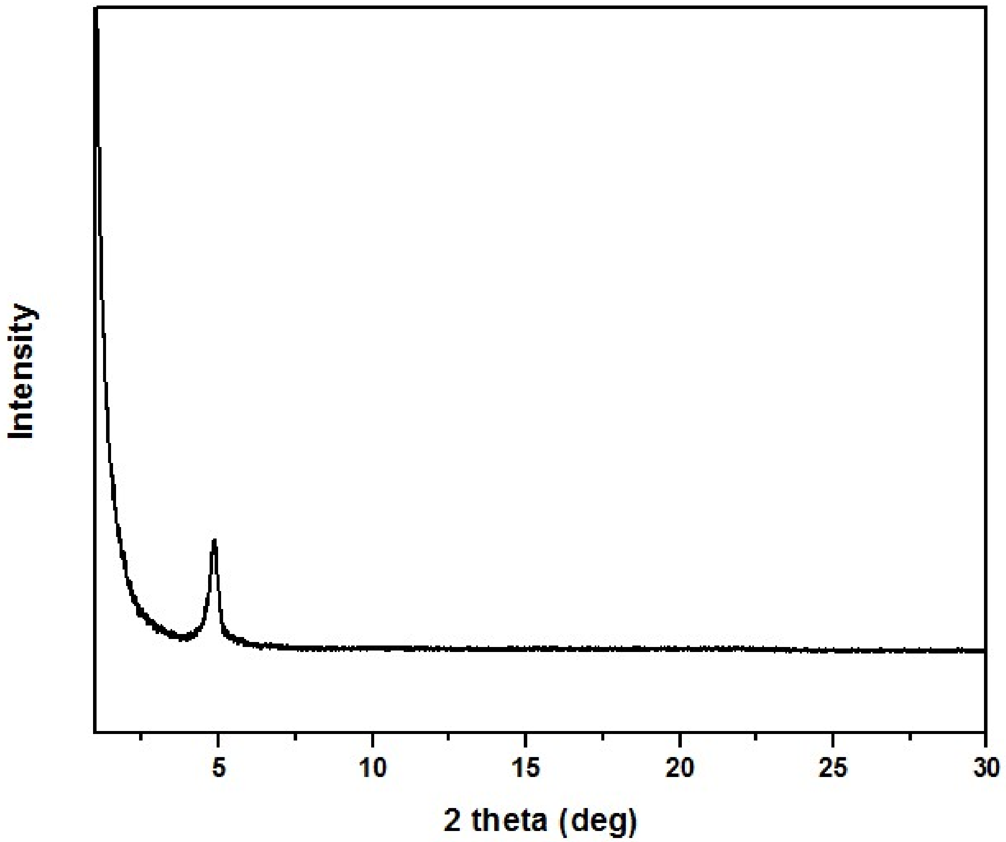

Figure S19. XRD of RICE-2 after 5 times recycling.

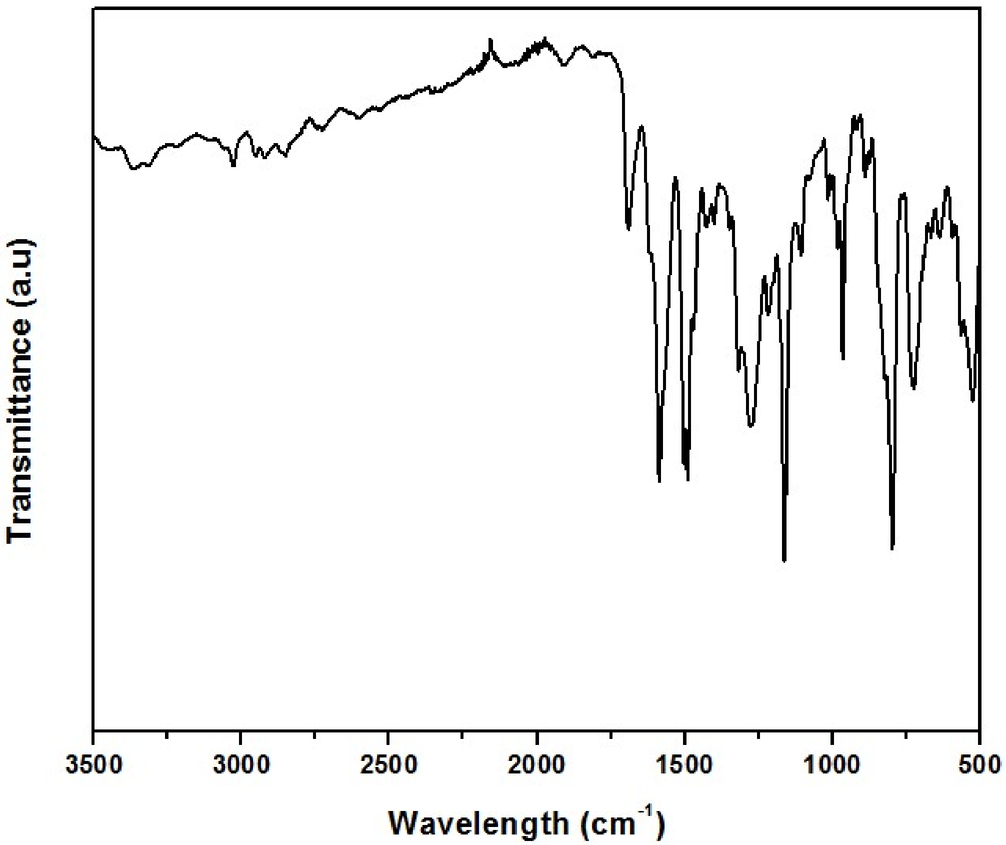

Figure S20. FT-IR of RICE-2 after 5 times recycling.

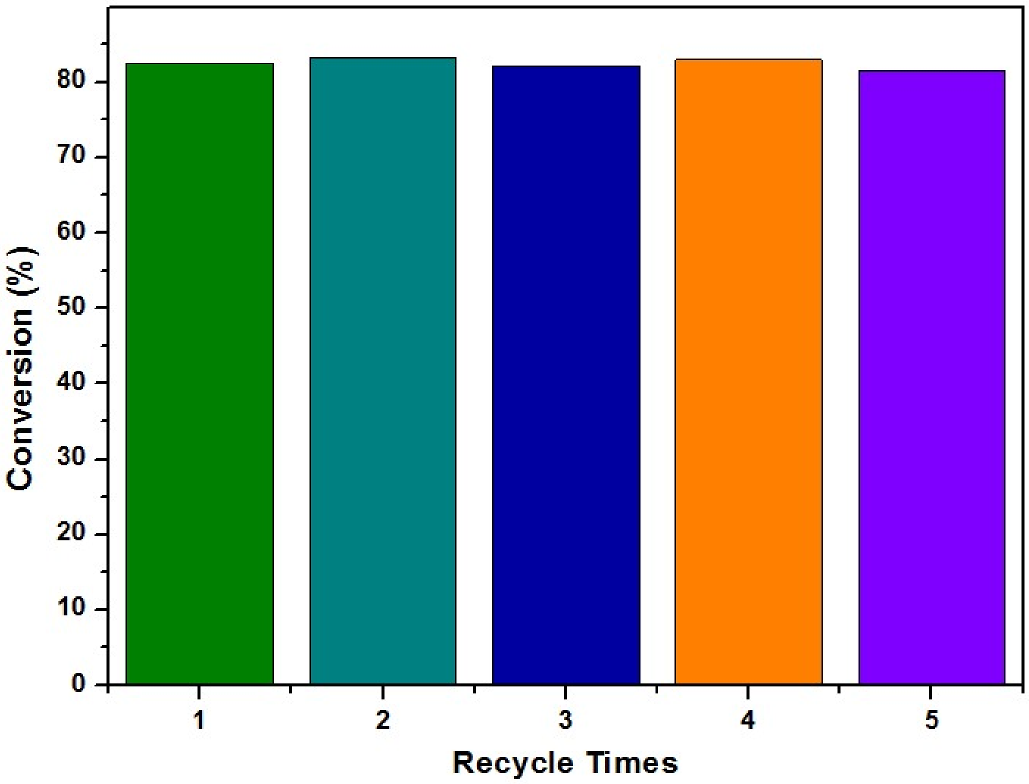

Figure S21. Monomer conversion of RICE-2 mediated PET-RAFT polymerization of MA in five independent polymerizations; The recycling test of RICE-2 was performed in DMF; DP=190. Reaction time = 17 h. The M_n and $Đ$ for all the reactions were summarized in Table S2.

Table S1. Control experiments for RICE-1 and RICE-2 catalyzed PET-RAFT polymerization of MA.[a]

Entry	PC	Light source	CTA[b]	Conv (%)[c]	$M_{n,GPC}$ (kDa)[d]	$M_{n,th}$ (kDa)[e]	$Đ$[d]
1	RICE-1	No	BTPA	0	/	/	/
2	RICE-2	No	BTPA	0	/	/	/
3	/	Blue	BTPA	3.2	/	/	/
4	RICE-1	Blue	/	0	/	/	/
5	RICE-2	Blue	/	0	/	/	/
6	TTAP	Blue	BTPA	1.2	/	/	/
7[f]	RICE-2	Blue	BTPA	70.9	22.1	11.8	1.37

a. General conditions: DP = 190. COF loading = 1mg/ml. Reactions were performed in DMF for 17 hours. For blue LED used for entries 3-6, light intensity is 15 mW/cm^2, λ_{max} is 460 nm. b. CTA stands for chain transfer agents c. Conversion was measured by ^{1}H NMR. d. M_n measured by GPC in tetrahydrofuran, based on linear polystyrene as the calibration standard.e. $M_{n,th}$ = DP × conversion × MW (monomer) + MW (initiator). f. Reaction is open to air.

Table S2. Monomer conversion, M_n and *Đ* of each recycling test in Figure S21.

Recycle times	Conv(%)	M_n (kDa)	*Đ*
1	82.5	13.7	1.07
2	83.2	13.9	1.10
3	82.1	14.5	1.11
4	83.0	13.6	1.09
5	81.5	14.0	1.14

Table S3. Compare the recyclability of COF(RICE-2) versus MOF-525 (Zn) in PET-RAFT polymerization.

Recycle times	Conv(%)- RICE 2	Conv(%)- MOF-525 (Zn)	*Đ*- RICE 2	*Đ*- MOF-525 (Zn)
1	82.5	81 ± 1.8	1.07	1.18
2	83.2	83 ± 2.4	1.10	1.15
3	82.1	80 ± 2.2	1.11	1.15
4	83.0	84 ± 2.6	1.09	1.16
5	81.5	80 ± 3.0	1.14	1.14

Note: The recyclability data of MOF-525 (Zn) was obtained from Angew.Chem. Int.Ed. 2021, 60,5489 –5496.

Table S4. Fractional atomic coordinates for RICE-1

Space group: *P222*			
a = 31.07, b = 26.31 Å, c = 4.39 Å; α = β = γ = 90°			
Atom	x	y	z
C1	0.52135	4.15806	8.52978
C2	0.53451	4.10777	8.52892
C3	0.59106	4.04098	8.5148
C4	0.57803	4.0923	8.54063
N5	0.71757	4.23473	8.58578
C6	0.6798	4.16158	8.78315
C7	0.68061	4.20242	8.58158
C8	0.64627	4.20931	8.37836
C9	0.61349	4.17333	8.36255
C10	0.61235	4.13212	8.56543
C11	0.64531	4.12763	8.78261
C12	0.95943	4.44117	10.49864
C13	0.95752	4.3956	10.33024
C14	0.92024	4.36605	10.32193
C15	0.88388	4.38166	10.48264
C16	0.88547	4.4256	10.65806
C17	0.92324	4.45374	10.67141
C18	0.84383	4.35307	10.46138
C19	0.839	4.30775	10.62359
C20	0.80041	4.28091	10.60577
C21	0.76682	4.29881	10.42037
C22	0.77191	4.34419	10.25794
C23	0.81006	4.37166	10.28259
C24	0.72673	1.27013	0.39053
C25	0.63369	1.02568	0.50141
N26	0.5	1.07658	0.5
C27	1	1.52677	0.5
N28	0.56462	1	0.5

Table S5. Fractional atomic coordinates for RICE-2.

Space group: *P222*			
a = 26.00, b = 26.00 Å, c = 4.42 Å; α = β = γ = 90°			
Atom	x	y	z
C1	0.52537	4.16089	8.53768
C2	0.541	4.10987	8.54439
C3	0.60791	4.04135	8.53471
C4	0.59259	4.09343	8.57748
N5	0.77837	4.20763	8.64269
C6	0.71761	4.1487	8.87142
C7	0.72678	4.18846	8.66727
C8	0.68686	4.20466	8.47468
C9	0.64178	4.17564	8.45372
C10	0.6342	4.13236	8.64032
C11	0.67108	4.12207	8.86496
C12	0.79692	1.23136	0.40971
C13	0.65887	1.02582	0.51648
C14	0.85124	1.75447	0.59828
C15	0.86659	1.71528	0.79402
C16	0.91697	1.69825	0.78997
C17	0.95346	1.7195	0.56817
C18	0.93816	1.76182	0.3953
C19	0.88742	1.77913	0.41034
C20	0.95562	1.60861	0.41809
C21	0.95588	1.5553	0.41303
C22	1	1.52821	0.5
C23	1	1.63629	0.5
N24	1	1.69201	0.5
N25	0.5	1.07852	0.5
N26	0.57631	1	0.5

Reference

(1) Valiev, M.; Bylaska, E. J.; Govind, N.; Kowalski, K.; Straatsma, T. P.; Van Dam, H. J. J.; Wang, D.; Nieplocha, J.; Apra, E.; Windus, T. L.; de Jong, W. A. NWChem: A Comprehensive and Scalable Open-Source Solution for Large Scale Molecular Simulations. *Comput. Phys. Commun.* **2010**, *181* (9), 1477–1489. https://doi.org/10.1016/j.cpc.2010.04.018.

(2) Becke, A. D. Density-Functional Exchange-Energy Approximation with Correct Asymptotic Behavior. *Phys. Rev. A* **1988**, *38* (6), 3098–3100. https://doi.org/10.1103/PhysRevA.38.3098.

(3) Lee, C.; Yang, W.; Parr, R. G. Development of the Colle-Salvetti Correlation-Energy Formula into a Functional of the Electron Density. *Phys. Rev. B* **1988**, *37* (2), 785–789. https://doi.org/10.1103/PhysRevB.37.785.

(4) Dunning, T. H. Gaussian Basis Sets for Use in Correlated Molecular Calculations. I. The Atoms Boron through Neon and Hydrogen. *J. Chem. Phys.* **1989**, *90* (2), 1007–1023. https://doi.org/10.1063/1.456153.

(5) Woon, D. E.; Dunning, T. H. Gaussian Basis Sets for Use in Correlated Molecular Calculations. III. The Atoms Aluminum through Argon. *J. Chem. Phys.* **1993**, *98* (2), 1358–1371. https://doi.org/10.1063/1.464303.

(6) Kresse, G.; Furthmüller, J. Efficient Iterative Schemes for Ab Initio Total-Energy Calculations Using a Plane-Wave Basis Set. *Phys. Rev. B* **1996**, *54* (16), 11169–11186. https://doi.org/10.1103/PhysRevB.54.11169.

(7) Kresse, G.; Furthmüller, J. Efficiency of Ab-Initio Total Energy Calculations for Metals and Semiconductors Using a Plane-Wave Basis Set. *Comput. Mater. Sci.* **1996**, *6* (1), 15–50. https://doi.org/10.1016/0927-0256(96)00008-0.

(8) Perdew, J. P.; Burke, K.; Ernzerhof, M. Generalized Gradient Approximation Made Simple. *Phys. Rev. Lett.* **1996**, *77* (18), 3865–3868. https://doi.org/10.1103/PhysRevLett.77.3865.

(9) Blöchl, P. E. Projector Augmented-Wave Method. *Phys. Rev. B* **1994**, *50* (24), 17953–17979. https://doi.org/10.1103/PhysRevB.50.17953.

(10) Kresse, G.; Joubert, D. From Ultrasoft Pseudopotentials to the Projector Augmented-Wave Method. *Phys. Rev. B* **1999**, *59* (3), 1758–1775. https://doi.org/10.1103/PhysRevB.59.1758.

(11) Grimme, S.; Antony, J.; Ehrlich, S.; Krieg, H. A Consistent and Accurate Ab Initio Parametrization of Density Functional Dispersion Correction (DFT-D) for the 94 Elements H-Pu. *J. Chem. Phys.* **2010**, *132* (15), 154104. https://doi.org/10.1063/1.3382344.

(12) Monkhorst, H. J.; Pack, J. D. Special Points for Brillouin-Zone Integrations. *Phys.*

Rev. B **1976**, *13* (12), 5188–5192. https://doi.org/10.1103/PhysRevB.13.5188.
(13) Verma, P.; Truhlar, D. G. HLE16: A Local Kohn–Sham Gradient Approximation with Good Performance for Semiconductor Band Gaps and Molecular Excitation Energies. *J. Phys. Chem. Lett.* **2017**, *8* (2), 380–387. https://doi.org/10.1021/acs.jpclett.6b02757.
(14) Heyd, J.; Peralta, J. E.; Scuseria, G. E.; Martin, R. L. Energy Band Gaps and Lattice Parameters Evaluated with the Heyd-Scuseria-Ernzerhof Screened Hybrid Functional. *J. Chem. Phys.* **2005**, *123* (17), 174101. https://doi.org/10.1063/1.2085170.
(15) John P. Perdew and Mel Levy. Physical Content of the Exact Kohn-Sham Orbital Energies: Band Gaps and Derivative Discontinuities. Phys. Rev. Lett. 51, 1884; **1983;** http://link.aps.org/doi/10.1103/PhysRevLett.51.1884
(16) Toroker, M. C.; Kanan, D. K.; Alidoust, N.; Isseroff, L. Y.; Liao, P.; Carter, E. A. First Principles Scheme to Evaluate Band Edge Positions in Potential Transition Metal Oxide Photocatalysts and Photoelectrodes. *Phys. Chem. Chem. Phys.* **2011**, *13* (37), 16644–16654. https://doi.org/10.1039/C1CP22128K.
(17) Trasatti, S. The absolute electrode potential: an explanatory note (Recommendations 1986). *Pure Appl. Chem.* **1986**, *58* (7), 955–966. https://doi.org/10.1351/pac198658070955.
(18) Wang, X.; Chen, L.; Chong, S. Y.; Little, M. A.; Wu, Y.; Zhu, W.-H.; Clowes, R.; Yan, Y.; Zwijnenburg, M. A.; Sprick, R. S.; Cooper, A. I. Sulfone-Containing Covalent Organic Frameworks for Photocatalytic Hydrogen Evolution from Water. *Nat. Chem.* **2018**, *10* (12), 1180–1189. https://doi.org/10.1038/s41557-018-0141-5.
(19) Butler, K. T.; Hendon, C. H.; Walsh, A. Electronic Chemical Potentials of Porous Metal–Organic Frameworks. *J. Am. Chem. Soc.* **2014**, *136* (7), 2703–2706. https://doi.org/10.1021/ja4110073.
